# Differential Proteomic Analysis of Human Erythroblasts Undergoing Apoptosis Induced by Epo-Withdrawal

**DOI:** 10.1371/journal.pone.0038356

**Published:** 2012-06-18

**Authors:** Stéphanie Pellegrin, Kate J. Heesom, Timothy J. Satchwell, Bethan R. Hawley, Geoff Daniels, Emile van den Akker, Ashley M. Toye

**Affiliations:** 1 School of Biochemistry, Medical Sciences Building, University Walk, Bristol, United Kingdom; 2 Proteomics Facility, University of Bristol, University Walk, Bristol, United Kingdom; 3 Bristol Institute for Transfusion Sciences, NHS Blood and Transplant, Filton, Bristol, United Kingdom; 4 Department of Hematopoiesis, Sanquin Research, Amsterdam, The Netherlands; Institut national de la santé et de la recherche médicale (INSERM), France

## Abstract

The availability of Erythropoietin (Epo) is essential for the survival of erythroid progenitors. Here we study the effects of Epo removal on primary human erythroblasts grown from peripheral blood CD34^+^ cells. The erythroblasts died rapidly from apoptosis, even in the presence of SCF, and within 24 hours of Epo withdrawal 60% of the cells were Annexin V positive. Other classical hallmarks of apoptosis were also observed, including cytochrome c release into the cytosol, loss of mitochondrial membrane potential, Bax translocation to the mitochondria and caspase activation. We adopted a 2D DIGE approach to compare the proteomes of erythroblasts maintained for 12 hours in the presence or absence of Epo. Proteomic comparisons demonstrated significant and reproducible alterations in the abundance of proteins between the two growth conditions, with 18 and 31 proteins exhibiting altered abundance in presence or absence of Epo, respectively. We observed that Epo withdrawal induced the proteolysis of the multi-functional proteins Hsp90 alpha, Hsp90 beta, SET, 14-3-3 beta, 14-3-3 gamma, 14-3-3 epsilon, and RPSA, thereby targeting multiple signaling pathways and cellular processes simultaneously. We also observed that 14 proteins were differentially phosphorylated and confirmed the phosphorylation of the Hsp90 alpha and Hsp90 beta proteolytic fragments in apoptotic cells using Nano LC mass spectrometry. Our analysis of the global changes occurring in the proteome of primary human erythroblasts in response to Epo removal has increased the repertoire of proteins affected by Epo withdrawal and identified proteins whose aberrant regulation may contribute to ineffective erythropoiesis.

## Introduction

Red blood cell production in the bone marrow is maintained by a delicate balance between erythroid cell proliferation, differentiation and apoptosis. This process is regulated by Erythropoietin (Epo), Stem Cell Factor (SCF) and glucocorticoids [1], [2]. Epo is a 34 kD glycoprotein produced primarily by the kidney and its production increases under hypoxic conditions [3]. It is essential for erythropoiesis [4] and the availability of Epo is known to facilitate the survival of erythroblasts during the Epo-dependent stage of erythropoiesis [5]. Epo acts by binding to its cognate receptor, the single transmembrane erythropoietin receptor (EpoR) [6]. EpoR lacks kinase activity but Epo binding triggers the activation of the Janus family protein tyrosine kinase 2 (JAK2) [7], which in turn phosphorylates tyrosine residues in EpoR, creating docking sites for intracellular signalling proteins such as phosphatidylinositol 3-kinase [8], SHP1 [9] and STAT5 [10]. These events lead to the activation of multiple signal transduction pathways and specific gene expression that result in the survival, proliferation, and differentiation of erythroblasts [11].

During homeostatic bone marrow erythropoiesis 16% of the erythroblasts die of apoptosis but this level of apoptosis is reduced by increased Epo [12]. Determining the molecular mechanisms behind the action of Epo is essential for our understanding of erythropoiesis in the bone marrow, thereby helping to efficiently reproduce erythropoiesis *in vitro*. It is also important for the development of novel erythropoiesis-stimulating agents and for understanding Epo’s cytoprotective action on other cell types [13]. It is also relevant to human disease since apoptotic mechanisms are implicated in the development of anaemia in myelodysplasia [12]. Understanding how Epo withdrawal induces apoptosis may also help improve apoptosis-inducing treatments of erythroid and non-erythroid leukaemia and identify the signalling pathways important for leukemic progression of specific leukemic clones.

Several molecular pathways involved in the induction of apoptosis in response to Epo withdrawal have been identified. For instance, studies on mice have shown that Epo inhibits pro-apoptotic Bim [14] and Bad [15] and induces anti-apoptotic SERPINA-3G and TRB3 [16]. In primary human erythroblasts, Epo inhibits pro-apoptotic GSK3 beta [17]. In addition, chaperone proteins play an important role in human erythroblast cell survival with Hsp70 preventing the transcription factor Gata-1 from being cleaved by Caspase 3 [18] and inhibiting the nuclear import of Apoptosis-inducing Factor (AIF) [19]. Another chaperone protein Mortalin has also been identified as a mediator of Epo signalling [20].

To further our understanding of how Epo withdrawal induces apoptosis, we adopted a 2 Dimensional fluorescence difference gel electrophoresis (2D DIGE) proteomics approach coupled with mass spectrometry to compare the proteomes of expanding erythroblasts with that of erythroblasts undergoing apoptosis due to Epo removal. Using this methodology we identified in an unbiased fashion, novel key reproducible alterations in the proteome of primary human erythroblasts +/−Epo. In particular, our results highlight that within 12 hours of Epo withdrawal, several multi-functional proteins are cleaved, including SET, RPSA, Hsp90 and 14-3-3- proteins. The proteolysis of proteins pivotal to many pro-survival cellular signalling cascades may be vital to ensure that the cell enters apoptotic cell death, and interestingly, aberrant regulation of these proteins is already known to occur in human diseases.

## Materials and Methods

### Erythroid Cell Culture

Waste peripheral blood from anonymous donors was provided with written informed consent for research use given in accordance with the Declaration of Helsinki (NHSBT, Filton, Bristol). The research into the mechanisms of erythropoiesis was reviewed and approved by the Southmead Research Ethics committee 08/05/2008 REC Number 08/H0102/26. Mononuclear cells (PBMCs) isolated from waste peripheral blood were washed in PBS, and the CD34+ cells isolated using anti-CD34^+^-ligated magnetic beads and the Magnetic Activated Cell Sorting system (MiniMACS) according to the manufacturer’s instructions (Miltenyi Biotech, UK). In order to minimise changes due to donor variation, erythroblasts were expanded from four different donors using culture conditions as described previously [21], [22]. For the first 4 days, cells were maintained in Stemspan (Stemcell Technologies) supplemented with 2 U/ml Epo (NeoRecormon, Roche), 10 ng/ml recombinant SCF (R&D Systems), 1 µM Dexamethasone (Sigma), 1∶200 cholesterol-rich Lipids (Sigma), 1 ng/ml IL-3 (R&D Systems), and Penicillin/Streptomycin (Sigma). Cells were then transferred to expansion medium ESDL which was identical except for the omission of IL-3. For comparison of the effects of Epo removal, CD34^+^ derived erythroblasts at day 9 (i.e. 4 days in ESDL+IL-3 followed by 5 days in ESDL only) were washed three times in PBS, seeded at 1.2×10^6^ cells/ml in fresh expansion medium in ESDL or SDL (expansion medium lacking EPO) and cultured for another 6 hour, 12 hour or 24 hour, as indicated. To obtain cell lysates for Western blotting and 2D-DIGE analysis, the cells were harvested by centrifugation, washed once with PBS, snap frozen in liquid nitrogen and stored at -80°C until further processing.

### Flow Cytometry

0.2−0.5×10^6^ cultured erythroid progenitors were washed in ice-cold PBS containing 1% (w/v) BSA (PBS-1%BSA) and incubated with the primary antibody for 1 hour. Primary antibodies used include BRIC6 (Band 3, IBGRL, Bristol, UK), BRIC 256 (Glycophorin A, IBGRL, Filton, Bristol), anti-Fas (CD95; Monoclonal antibody LOB 3/17, Serotec), anti-FasL (CD178; monoclonal antibody 10F2, Serotec) and suitable mouse IgG control antibodies (Dako). Primary antibodies were washed in ice-cold PBS-1% BSA and rabbit anti-mouse RPE-conjugated antibodies (Dako) were added for 30 min in the dark at 4°C. Directly conjugated antibodies used were anti-c-kit/CD117 (RPE-conjugated, BD Pharmingen, 555714) and anti-CD71 (RPE or APC-conjugated, BD Pharmingen, 555537). To measure apoptotic cell death, cultured erythroblasts were labelled with Annexin V-FITC, together with Propidium Iodide according to the manufacturer’s instructions (Arcus Biologicals). To measure mitochondrial membrane potential (ΔΨ), tetramethylrhodamine ethyl ester perchlorate (TMRE, Sigma) was used. Erythroblasts were washed in PBS, resuspended in PBS containing 25 nM TMRE. To quantify caspase activation by flow cytometry, Caspase-3, Caspase-8 and Caspase 9 detection kits were used according to the manufacturer’s instructions (Calbiochem). Fluorescent signals were measured using a Coulter EPICS XL-MCL flow cytometer (Beckman Coulter, HighWycombe, UK) or a FACS CantoII-F60 machine (BD Biosciences). All data was analysed using the Flowjo 7.2.5 software (Flowjo, Ashland, OR, USA).

### Cytospins

2.5×10^4^ cells were cytospun onto glass slides, fixed in methanol and stained with May Grünwald/Giemsa stains according to the manufacturer’s protocol. Images were taken with an Olympus CX31 microscope coupled to an Olympus LC20 camera using a 50x (0.75NA) lens and processed using Adobe Photoshop 9.0 (Adobe).

### Subcellular Fractionation to Detect Cytochrome C Release into the Cytosol

Cytochrome c release into the cytosol was assessed as previously described [23]. Cells (2×10^6^) were washed in PBS, resuspended in 50 µl of buffer (140 mM mannitol, 46 mM sucrose, 50 mM KCl, 1 mM KH_2_PO_4_, 5 mM MgCl_2_, 1 mM EGTA, 5 mM Tris, pH 7.4) supplemented with a mixture of protease inhibitors (Complete Mini-EDTA Free, Roche) and digitonin at a final concentration of 40 mg/ml. Cells were permeabilised on ice for 10 min and centrifuged at 12,000×*g* for 10 min at 4°C. Supernatant and pellet fractions were subjected to Western blot analysis.

### Western Blotting

5x10^6^ cells were lysed for 10 min on ice in lysis buffer (20 mM Tris-HCl, pH 8.0, 137 mM NaCl, 10 mM EDTA, 100 mM NaF, 1% (v/v) Nonidet P-40, 10% (v/v) glycerol, 10 mM Na_3_VO_4_, 2 mM PMSF and protease inhibitors, Calbiochem). Protein concentration determined by Lowry assay (Bio-Rad). Lysates were separated by SDS-PAGE and immunoblotted. Primary antibodies used (with catalog numbers in brackets) were Caspase 8 (1C12, 9746), Caspase 9 (9502), cleaved Caspase 3 (9664), Hsp90beta (5087) and Lamin A/C (2032) from Cell Signalling Technology; Hsp90alpha (mAb 9D2, SPA-840) from Enzo/Stressgen; Actin (sc-1616 rabbit), RPSA (Laminin-R (16), sc-101517) and SET (I2PP2A, sc-5655) from Santa Cruz; Cytochrome C (Clone 7H8.2C12, 556443) from BD Pharmingen; Bax (anti-Bax NT, 06–499), 14-3-3 beta (AB9730), 14-3-3 epsilon (clone CG31-2B6, 05-639) and 14-3-3 gamma (AB9734) from Millipore/Upstate Cell Signalling and Hsc70 (ab19136) from Abcam.

### Immunofluorescence Microscopy

1.5-2×10^5^ erythroblasts were left to adhere on poly-L-lysine coated coverslips (mol wt 70,000–150,000, 0.01% w/v solution; Sigma) for 30 min at 37°C, 5% CO_2_ before fixation using 4% formaldehyde (TAAB Laboratories Ltd, Aldermaston, England, UK) in PBS for 15 min. For some experiments, 100 nM MitoTracker® Red CMXRos (Invitrogen) was included. Cells were washed in PBS and then permeabilised with 0.2% (w/v) Triton X-100 in PBS for 5 min or ice-cold methanol for 1 min. Cells were washed in PBS, blocked for 20 min in PBS-4%BSA and incubated for 1 hour in primary antibodies diluted in PBS-1% BSA. After further washes in PBS, cells were incubated for 1 hour with secondary antibodies in PBS-1% BSA. After 3×5 min washes in PBS, cells were stained with Hoechst (2 mg/ml, Invitrogen) for 5 min, washed and mounted over MOWIOL 4-88 (Calbiochem) containing 0.6% 1,4-diazabicyclo-(2.2.2)octane (DABCO, Sigma) as an anti-photobleaching agent. Confocal microscopy was performed using a Leica AOBS SP2 confocal microscope (x63/1.4 oil-immersion objective). A serial Z stack at 0.5 µm intervals was taken and a projected image produced using Leica software. The primary antibodies used include Bak-NT and Bax-NT (Upstate Cell signalling), and BRIC256 (Glycophorin A). The secondary antibodies were Goat anti-Mouse or anti-Rabbit, Alexa 488 or Alexa 594 (Invitrogen).

### Sample Preparation for 2D- DIGE

Cell pellets (4.5-8×0^6^ erythroid progenitors per pellet) were resuspended in 2D lysis buffer (7 M urea, 2 M thiourea, 4% (w/v) CHAPS), sonicated in a water bath for 15 min and incubated for 2 hour at room temperature with intermittent vortexing. Solubilised samples were then precipitated using a 2-D Clean-Up Kit (GE Healthcare) according to the manufacturer’s instructions and the resulting pellets were resuspended to a concentration of between 5 and 10 mg/ml in DIGE lysis buffer (30 mM Tris, pH 8.5, 7 M Urea, 2 M Thiourea, 4% (w/v) CHAPS). 50 µg of each sample was labeled for DIGE analysis using fluorescent cyanine dyes according to the manufacturer’s guidelines (GE-Healthcare). In brief, samples were labeled using Cy3 or Cy5 N-hydroxysuccinamide (NHS) ester DIGE dyes freshly dissolved in anhydrous dimethylformamide by mixing 50 µg protein with 1 µL CyDye (400 pmol/µL). An internal standard was generated by pooling all samples in the experiment and labelling with a third dye, Cy2. In each case, the labelling reaction was allowed to proceed on ice in the dark for 30 min. The reaction was terminated by the addition of 10 nmol lysine and subsequent incubation on ice in the dark for an additional 10 min.

### 2D Gel Electrophoresis

Each Cy3- and Cy5-labelled sample pair was mixed with an aliquot of the Cy2-labelled internal standard and Destreak rehydration solution (GE Healthcare) containing 0.5% (v/v) IPG Buffer pH3-11NL added to give a total volume of 450 µL. This was loaded onto a 24 cm Immobiline DryStrip gel (pH 3–11 non-linear) by passive rehydration for a minimum of 12hour. Following rehydration, the DryStrip gel was transferred to an Ettan IPGPhor 3 system (GE Healthcare) and isoelectric focusing performed according to the manufacturer’s instructions (in brief, by applying 500 Volts for 1 hour, increasing to 1,000 Volts over 1 hour, and then to 10,000 Volts over 3 hours and held at 10,000V for a further 2.5 hour). After isoelectric focusing, strips were equilibrated in SDS equilibration buffer (50 mM Tris-HCl, pH 8.8, 6 M urea, 30% (v/v) glycerol, 2% (w/v) SDS, and 0.002% (w/v) bromphenol blue) containing 1% (w/v) DTT for 15 min at room temperature followed by a second incubation in SDS equilibration buffer containing 2.5% (w/v) iodoacetemide for 15 min at room temperature. After equilibration, strips were applied to 12.5% (w/v) SDS-PAGE gels and run at 5 mA per gel for 1 hour, 8 mA per gel for an additional hour and then at 13 Watts/gel until completion on an Ettan DALT-6 separation unit (GE Healthcare). Each gel was scanned at three separate wavelengths using a Typhoon 9400 variable mode imager (GE Healthcare) to generate Cy3, Cy5 and Cy2 images. Determination of protein spot abundance and analysis of protein expression changes between samples was conducted on DeCyder V6.5 software (GE Healthcare). Spots which were present in all samples and which showed a change in average ratio of +1.3 or -1.3 fold with a *t*-test of *p*<0.05 were chosen for identification by mass spectrometry. Analysis of the DIGE gels using the DeCyder software identified 2437 spots in the master gel; of these, 1569 were reproducibly detected and quantified in all 4 gels used in the experiment. Only spots that were detected in all 4 gels (i.e. in all 4 independent DIGE experiments) were selected for identification by mass spectrometry.

### Proteolytic Digestion and Mass Spectrometry

For preparative gels, pooled samples were generated by combining 100 µg of each SDL or ESDL sample prior to DIGE labelling. Following 2D-PAGE (as above), the resulting gels were stained using SYPRO® Ruby total protein stain (Invitrogen) and visualised using a Typhoon 9400 variable mode imager (GE Healthcare). Spots selected for Mass spectrometry were picked using the Investigator ProPic Automated 2-D spot picker and digested with trypsin using the ProGest automated digestion unit (both from Digilab UK Ltd). The resulting peptides were then subjected to Mass Spectrometry. Mass spectra were recorded in positive ion mode on an Applied Biosystems 4700 MALDI mass spectrometer. MS spectra were recorded in reflector mode. For MSMS analysis the top 5 most intense, non-tryptic, precursors were selected for fragmentation by collision induced dissociation. Neither baseline subtraction nor smoothing were applied to recorded spectra. MS and MSMS data were analyzed using GPS Explorer 3.5 (Applied Biosystems). MS peaks were filtered with a minimum signal to noise ratio of 35 and to exclude masses derived from trypsin autolysis. MSMS peaks were filtered to exclude peaks with a signal to noise ratio less than 35 over a mass range of 50Dalton to 20Dalton below the precursor mass. The mass spectral data for each spot was subjected to a combined analysis using the MASCOT algorithm (Matrix Science) against the NCBInr Human database. The combined analysis uses the initial MS spectra as a peptide mass fingerprint with supporting sequence data provided by up to 5 MSMS spectra per spot. A maximum number of missed cleavages of 1 and a charge state of +1 were assumed for precursor ions. A precursor tolerance of 100 ppm and an MSMS fragment tolerance of 0.15Dalton were used in the database search. Routinely, samples were analysed with methionine oxidation considered as a variable modification and carbamidomethylation of cysteine as a fixed modification.

### Protein Phosphorylation

To analyse changes in protein phosphorylation between control (ESDL) and test (SDL) samples, two 2D preparative gels were prepared as above containing pooled protein (100 µg each) of the 4 ESDL or SDL cultures. These were stained for phosphoproteins using Pro-Q Diamond phosphoprotein stain (Invitrogen) and imaged using a Typhoon 9400 Variable Mode Imager (GE Healthcare). Gels were then stained for total protein using SYPRO® Ruby protein gel stain (Invitrogen) and imaged again. Differences in the pattern of protein phosphorylation were identified using ImageQuant v5.2 software and the corresponding spots were excised from the SYPRO® Ruby stained gel and identified by mass spectrometry (as described above). The 12 hour SDL 2D PAGE gels were reproduced again in duplicate, using 400 µg protein loaded per gel from two further independent experiments. The equivalent spots were then subjected to Nano LC Mass Spectrometry. Briefly, selected spots were excised and subjected to in-gel tryptic digestion using a ProGest automated digestion unit (Digilab UK). The resulting peptides were fractionated using a Dionex Ultimate 3000 nanoHPLC system. In brief, peptides in 1% (v/v) formic acid were injected onto an Acclaim PepMap C18 nano-trap column (Dionex). After washing with 0.5% (v/v) acetonitrile 0.1% (v/v) formic acid peptides were resolved on a 250 mm × 75 µm Acclaim PepMap C18 reverse phase analytical column (Dionex) over a 120 min organic gradient with a flow rate of 300 nl min^−1^. Peptides were ionised by nano-electrospray ionisation at 2.0 kV using a stainless steel emitter with an internal diameter of 30 µm (Thermo Scientific). Tandem mass spectrometry analysis was carried out on a LTQ-Orbitrap Velos mass spectrometer (Thermo Scientific). The Nano LC was set to analyse the survey scans at 60,000 resolution and the top twenty ions in each duty cycle selected for MSMS in the LTQ linear ion trap. Data was acquired using the Xcalibar v2.1 software (Thermo Scientific). The raw data files were processed using Proteome Discoverer software v1.2 (Thermo Scientific) with searches performed against the SwissProt Human database (54523 entries) using the Mascot search engine v1.9 (Matrix Science) with the following criteria; peptide tolerance  = 10 ppm, trypsin as the enzyme, carbamidomethylation of cysteine as a fixed modification and oxidation of methionine and phosphorylation of serine, threonine and tyrosine as variable modifications. Individual ions with Mascot scores higher than 20 were used, making sure the average peptide scores of all identified proteins exceeded 20, a threshold commonly used for confident protein identification from tandem MS data [24]. The reverse database search option was enabled and all data was filtered to satisfy false discovery rate (FDR) of less than 5%.

## Results

### Apoptosis Occurs in Response to Epo Withdrawal in Cultured Primary Human Erythroblasts

**Figure 1:**
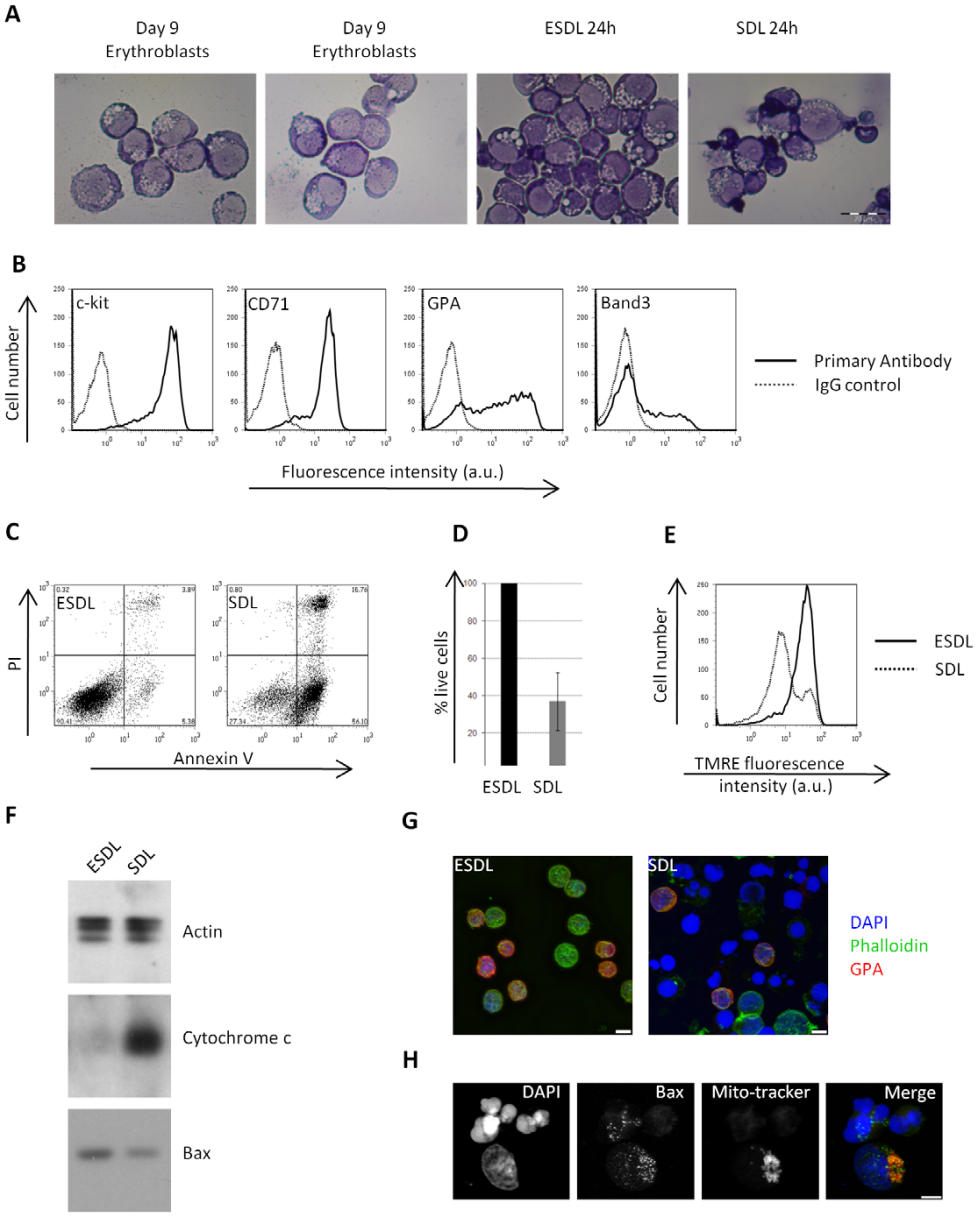
Epo removal induces apoptosis of erythroblasts. A) Cytospins of 2 separate erythroblast cultures obtained after 9 days in culture in ESDL medium and of erythroblasts after 24 hour in ESDL or SDL (scale bar is 20 µM). B) Flow cytometry analysis of cell surface markers expressed by erythroblasts after 8 days in culture in ESDL medium. FL2 fluorescence (x axis) versus cell number (y axis) of cells labelled with the isotype control antibody (dotted grey line) and antibodies against CD117/c-kit, CD71, GPA (BRIC256) and Band 3 (BRIC6) (thick black line). C) Flow cytometry analysis of Annexin V (FL1) and Propidium Iodide (PI, FL3) labelling of erythroblasts kept for 24 hour in ESDL or SDL. In this representative experiment, 90% of the cells kept in ESDL are alive (Annexin V and PI negative) compared to only 28% in SDL. D) Graph showing the average percentage of live erythroblasts kept for 24 hour in ESDL or SDL, normalised to the percentage of live cells in ESDL. After 24 hour, only 37% of the cells in SDL are live (AnV/PI negative, with a standard deviation of +/−15%, n = 15). E) Flow cytometry analysis of mitochondrial membrane potential (ΔΨ) using TMRE. In this representative experiment, the FL2 fluorescence for erythroblasts cultured for 24 hour in ESDL (thick black line) is overlayed with that of cells cultured for 24 hour in SDL (dotted grey line). The loss of TMRE fluorescence indicates a loss of mitochondrial membrane potential (ΔΨ). F) Cytochrome C release into the cytosol. Western blots against cytochrome c and total Bax were carried out on the cytosolic fraction depleted of all organelles, obtained from erythroblasts kept for 24 hour in ESDL or SDL. Beta actin was used as a loading control. G) Overlay projections of confocal images taken from erythroblasts grown for 24 hour in ESDL or SDL (blue: DAPI; green: 488 Phalloidin; red: GPA) showing that cells cultured for 24 hour in the absence of Epo have lost their plasma membrane integrity and have fragmented nuclei. H) Translocation of Bax to the mitochondria in cells kept for 24 hour in SDL. Overlay projections of confocal images taken from erythroblasts grown for 24 hour in SDL (blue: DAPI; green: Bax; red: Mito-tracker). Scale bar on images represents 5 µm.

Primary human erythroblasts were cultured from CD34^+^ cells isolated from human peripheral blood in presence of Epo, SCF, Dexamethasone and lipids (ESDL) [21]. This expansion medium (ESDL) allows CD34^+^ cells to expand and differentiate to the pro-erythroblast stage, whilst limiting pro-erythroblast terminal differentiation. During culture in ESDL, expanding erythroblasts become progressively GPA positive but maintain low or no expression of the early to late differentiation marker band 3 (Figure S1A). Importantly, throughout the ESDL culture conditions, the cells remained highly sensitive to Epo withdrawal (Figure S1B). The day 9 time point of expansion was chosen as this maximised the number of cells for our 2D DIGE experiments but limited the degree of spontaneous differentiation. At day 9 the majority of cells had the morphology of pro-erythroblasts (Figure 1A; counting 40 fields of view from each of two representative cultures; 2–4% pre-pro-erythroblasts, 79–80% pro-erythroblasts, and 16–19% basophilic erythroblasts) and are c-kit^+^ positive, CD71^high^, GPA^low^/^med^ and band 3^low/neg^ (Figure 1B and Figure S1A). The cells are also Fas positive but Fas ligand negative, which is consistent with them being immature erythroblasts [25] (Figure S2).

**Figure 2:**
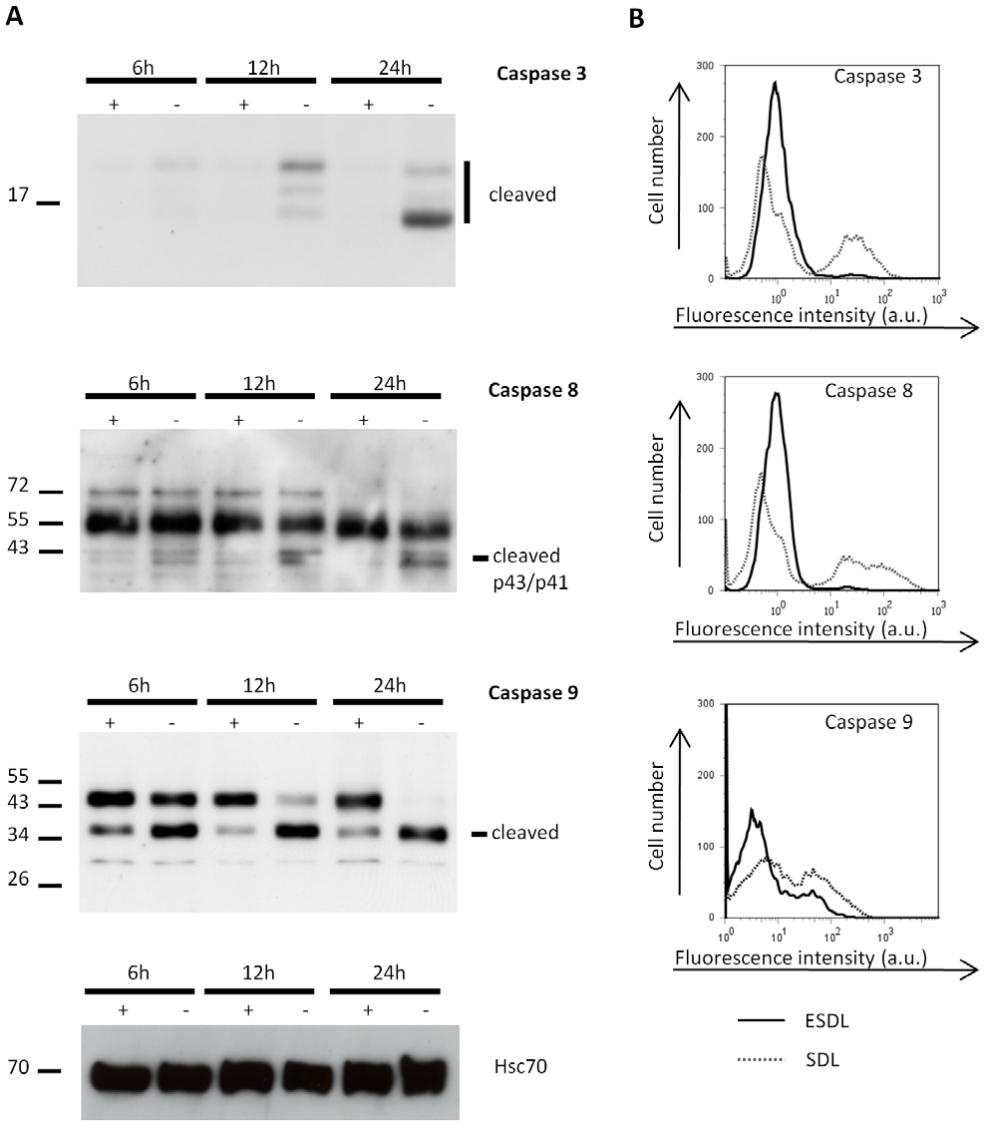
Caspase activation after Epo removal. Western blotting (A) of total cell lysates from one independent culture, harvested after 6 hour, 12 hour and 24 hour in ESDL (+Epo) and SDL (-Epo) using an antibody against cleaved caspase 3, caspase 9, caspase 8, and hsc70 was used as a loading control. 20 µg of protein lysate was loaded in each well (B) Flow Cytometry analysis of active caspase 8, active caspase 3 and active caspase 9 for ESDL (thick black line) grown cells and SDL (dotted grey line) after 24 hours. Active caspase 9 was detected on a separate culture from the caspase 3 and caspase 9 and using a different flow cytometer.

To monitor i) specific alterations in Epo signalling that cannot be compensated by SCF or dexamethasone and ii) the effect of Epo withdrawal on cellular processes, day 9 cells were either maintained in ESDL (+Epo) or SDL (no Epo). Apoptosis and loss of mitochondrial membrane potential was measured by flow cytometry using annexin V/PI and TMRE, respectively (Figure 1C,1D and 1E). After 24 hour of Epo withdrawal, 63% (+/−15%, n = 15) of the cells were Annexin V positive and a sharp decrease in mitochondrial potential was observed, indicative of apoptosis (Figure 1D and 1E). Loss of mitochondrial membrane potential was accompanied by cytochrome c release from the mitochondria into the cytosol (Figure 1F) and by translocation of cytosolic Bax to the mitochondria (Figure 1H). Epo removal further induced DNA condensation and nuclei fragmentation as well as reduced cortical actin and Glycophorin A staining (Figure 1G), indicating that these cells have lost the integrity of their plasma membrane. We also confirmed by Western blotting and flow cytometry that caspase 3, caspase 8 and caspase 9 were activated upon 24 hours of Epo removal (Figure 2) [26]. Furthermore, no Fas Ligand was detectable on pro-erythroblasts by flow cytometry after 24 hours Epo removal (Figure S2).

### 2D DIGE Analysis of Primary Human Erythroid Cells 12 Hour After Epo Withdrawal

#### Proteins identified with a change in average intensity ratio of >2

To identify global proteome alterations in erythroblasts after Epo removal, a 2D DIGE approach was adopted. Late apoptotic events involve the proteolysis of many proteins and signalling events not directly involved in the initial induction of apoptosis. After 12 hours in SDL (no Epo), the first signs of apoptosis were observed by flow cytometry as the cells become Annexin V positive but are not yet TMRE^low^ or PI^+ve^ (Figure S3). Therefore, to study the early events leading to apoptosis, rather than the later downstream events, we studied proteome changes after 12 hours of Epo removal by comparing the proteomes of erythroblasts kept in expansion medium (ESDL, 12 hours) with those switched to medium lacking Epo for 12 hours (SDL, 12 hours).

**Figure 3:**
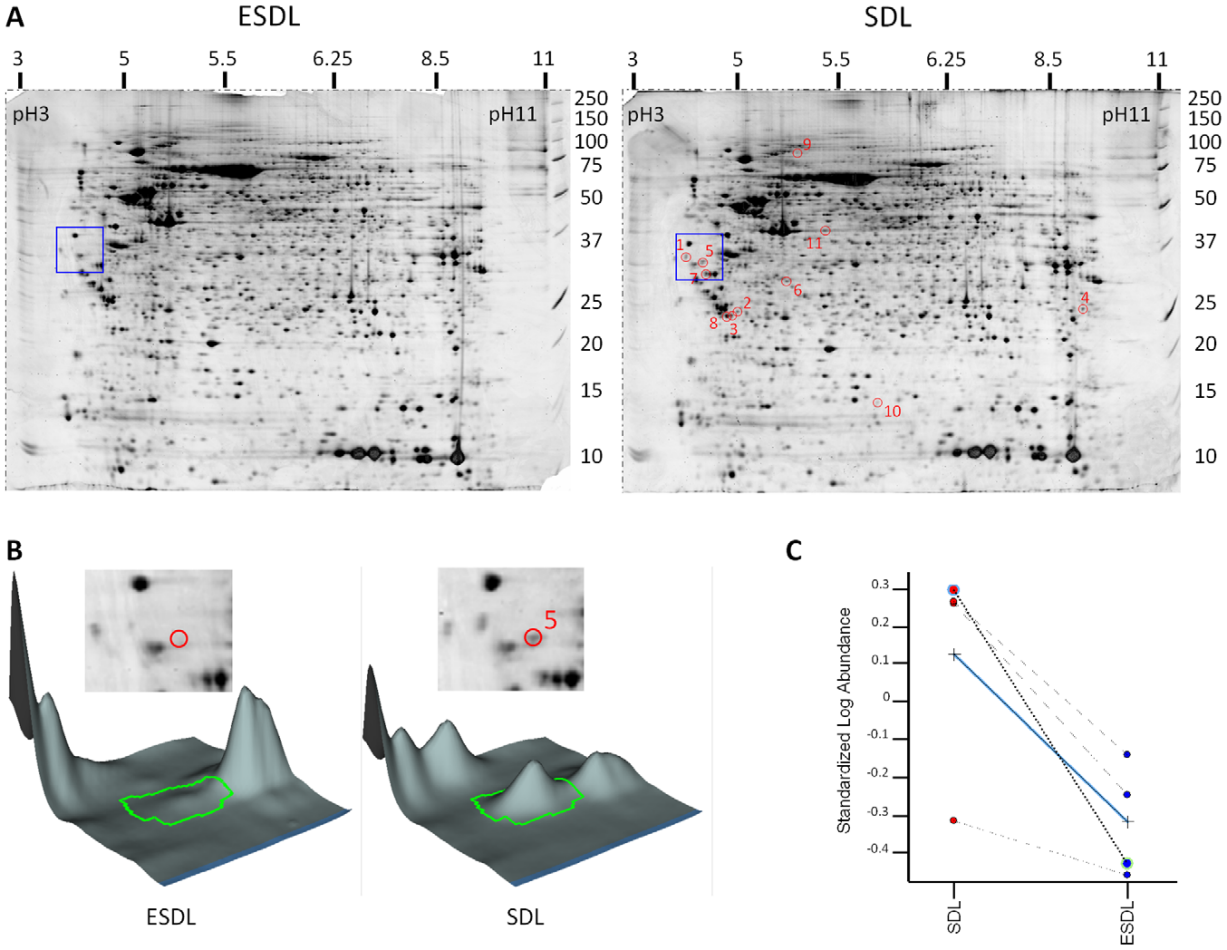
2D gels and spots identified after Epo removal. (A) 2D gel analysis of total cell lysates harvested after 12 hour in ESDL (+Epo) and SDL (-Epo). The 11 spots circled on the SDL 2D gel (right gel) were found to be consistently up-regulated in SDL (change of average ratio >2, t-test of p<0.05) when comparing the four SDL with the four ESDL 2D gels. These 11 spots are described in Table 1. The blue square depicts the region of the gel shown in Figure 3B. (B) Typical example of a protein differentially represented in the 2 culture conditions, ESDL and SDL. 3D views of spot 5 in Figure 3A and identified by mass spectrometry as a proteolytic fragment of Hsp90 alpha (see Table 1). The difference in intensity of spot 5 on one SDL and one ESDL gel is visible by eye on the 2D gels (spot marked with the red boundary) and on the corresponding 3D views. (C) Example graph showing the amount of protein for spot 5 in all 8 gels (4 SDL gels and 4 ESDL gels), together with the average ratios linked by the blue line.

**Figure 4:**
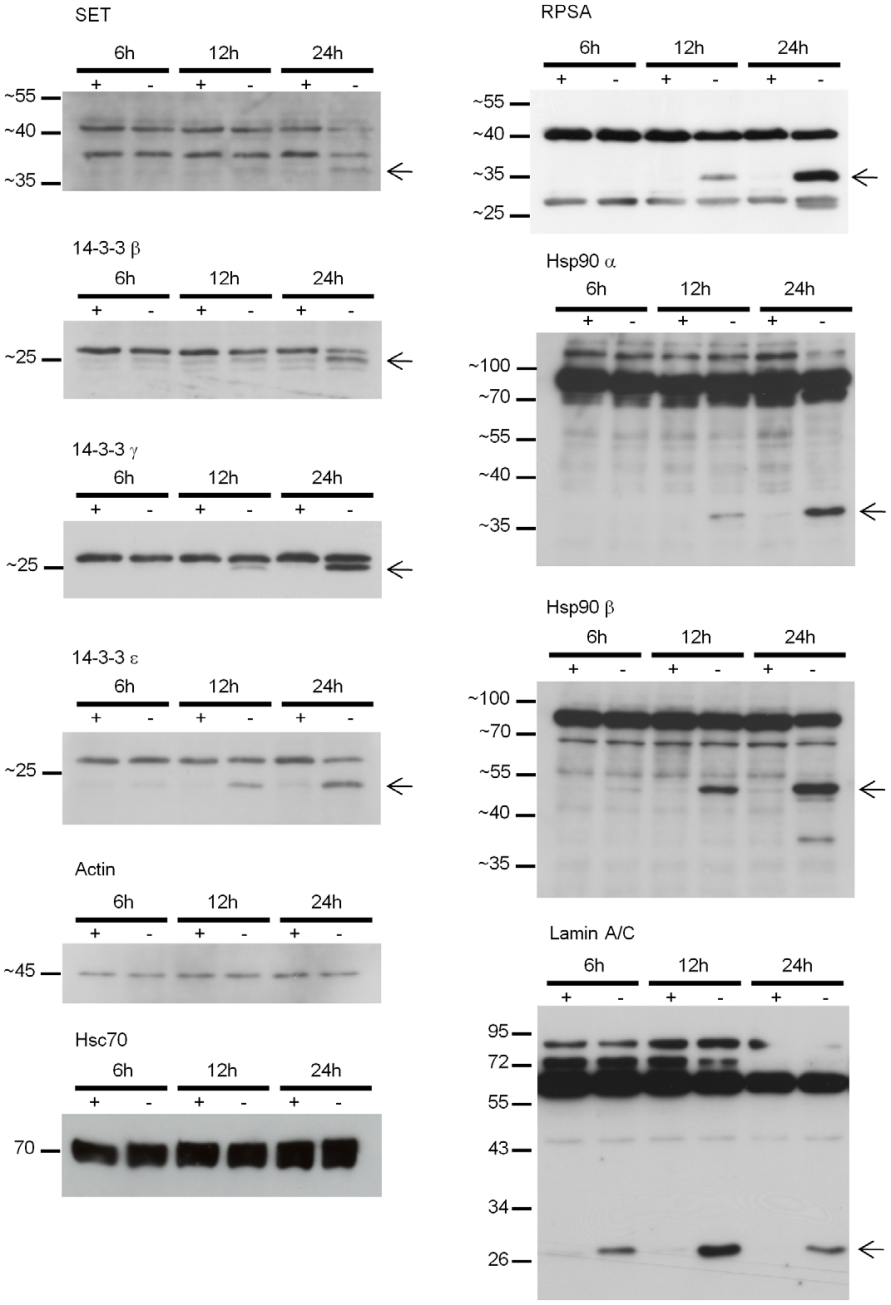
Western blot confirmation of protein proteolysis under SDL conditions. Western blotting of total cell lysates harvested from one independent culture after 6 hour, 12 hour and 24 hour in ESDL (+Epo) and SDL (-Epo) using antibodies against SET, 14-3-3 β, 14-3-3 γ, 14-3-3 ε, RPSA, Hsp90 isofoms alpha and beta. 20 µg of protein lysate was loaded per lane. Beta Actin and Hsc70 were used as loading controls. The arrows point to the smaller proteolytic fragments that occur in apoptotic erythroblasts.

By comparing 4 independent 2D DIGE experiments, 12 spots were consistently found to be up-regulated in SDL (apoptotic cells) with a change in average intensity ratio of >2 and a *t*-test of *p*<0.05. All 12 spots were picked from the gels (SDL, Figure 3A), and the identities of 11 of these spots were confirmed by mass spectrometry (Table 1 and Table S1). These include SET, 14-3-3 isoforms (beta, gamma and epsilon), Hsp90 alpha (Figure 3B) and beta, 40S ribosomal protein SA (RPSA) and non-muscle myosin heavy chain (myosin 9). Figure 3C illustrates the reproducibility of the observed alterations in abundance, showing the quantification of Hsp90 alpha isoform 2 (spot 5). For specific spots (e.g. spots 5 (Hsp90 alpha), 7 (Hsp90 beta) and 9 (Myosin 9)), the molecular weight of the spot picked for MS analysis was significantly smaller than the theoretical molecular weight of the full-length protein (Table 1), possibly as a result of caspase cleavage. Indeed, 9 out of the 11 proteins identified are known caspase substrates. Western blot analysis of total cell lysates from a different independent experiment validated the 2D DIGE proteomic results confirming the proteolysis of SET, 14-3-3 beta, 14-3-3 gamma, 14-3-3 epsilon, Hsp90 alpha, Hsp90 beta and RPSA upon Epo withdrawal (Figure 4).

**Table 1:** Proteins with altered abundance in SDL with a change in average ratio of >2 and a *t*-test of *p*<0.05.

Spot No.	t-test	Average ratio	Identified Proteins	Accesion number (Gene ID)	Molecular mass (kD)		p*I*		No of peptides matched	Mascot Protein Score (>66)	Precursor ion mass	MSMS Peptide sequence	Ion score
					E	T	E	T					
1	0.016	3.58	SET isoform 2	gi|170763498 (SET, 6418)	∼35	32	∼4.2	4	9	332	1063.6047	LRQPFFQK	41
											1208.6045	VEVTEFEDIK	72
											1840.8064	IDFYFDENPYFENK	107
											2195.021	EQQEAIEHIDEVQNEIDR	29
2	0.0074	3.55	14-3-3 gamma	gi|5726310 (YWHAG, 7532)	∼25	28.4	∼4.9	4.6	9	209	1205.6559	DSTLIMQLLR	4
											1261.6106	EHMQPTHPIR	27
											1643.7871	NVTELNEPLSNEER	101
3	0.0043	3.54	14-3-3 beta/alpha	gi|4507949 (YWHAB, 7529)	∼25	28	∼4.8	4.7	13	308	1205.6559	DSTLIMQLLR	17
											1598.7405	AVTEQGHELSNEER	108
											2159.0251	QTTVSNSQQAYQEAFEISK	68
4	0.025	3.27	heterogeneous nuclear ribonucleoproteins A2/B1 isoform A2	gi|4504447 (HNRNPA2B1, 3181)	∼25	36	∼9	8.7	15	367	1013.4434	GGNFGFGDSR	33
											1188.6471	IDTIEIITDR	33
											1377.6294	GGGGNFGPGPGSNFR	91
											1410.6873	YHTINGHNAEVR	58
5	0.037	3.03	heat shock protein HSP 90-alpha isoform 2	gi|154146191 (HSP90AA1, 3320)	∼35	84.6	∼4.3	4.9	12	497	1589.8759	ELHINLIPNKQDR	110
											1778.9475	HSQFIGYPITLFVEK	121
											2015.0443	VILHLKEDQTEYLEER	116
											2255.9587	HNDDEQYAWESSAGGSFTVR	90
6	0.025	2.73	40S ribosomal protein SA	gi|9845502 (RPSA, 3921)	∼29	32.8	∼5.25	4.7	15	500	1203.6481	FAAATGATPIAGR	81
											1698.8599	FTPGTFTNQIQAAFR	102
											1740.949	AIVAIENPADVSVISSR	133
											2081.0562	FTPGTFTNQIQAAFREPR	51
7	0.023	2.71	heat shock protein HSP 90-beta	gi|20149594 (HSP90AB1, 3326)	∼30	83.3	∼4.4	4.9	19	593	1194.6477	IDIIPNPQER	74
											1808.9581	HSQFIGYPITLYLEK	111
											2015.0443	VILHLKEDQTEYLEER	134
											2255.9587	HNDDEQYAWESSAGGSFTVR	161
8	0.00088	2.38	14-3-3 epsilon	gi|5803225 (YWHAE, 7531)	∼25	29.2	∼4.7	4.5	13	303	1205.6559	DSTLIMQLLR	30
											1256.5906	YLAEFATGNDR	96
											1384.6855	YLAEFATGNDRK	37
											1835.932	AASDIAMTELPPTHPIR	30
9	0.00045	2.19	Myosin 9	gi|12667788 (MYH9, 4627)	∼100	226.5	∼5.3	5.5	19	185	1155.6633	RGDLPFVVPR	44
											1869.9664	ANLQIDQINTDLNLER	57
											1949.9927	LQQELDDLLVDLDHQR	36
10	0.02	2.14	stathmin isoform a	gi|5031851 (STMN1, 3925)	∼15	17.3	∼5.75	5.9	1	81	1388.7532	ASGQAFELILSPR	75
11	0.019	2.05	eukaryotic initiation factor 4A-I	gi|4503529 (EIF4A1, 1973)	∼42	46.1	∼5.4	5.3	18	419	1068.5472	QFYINVER	27
											1634.8683	LQMEAPHIIVGTPGR	50
											1827.9387	GIYAYGFEKPSAIQQR	118
											2144.1345	GIDVQQVSLVINYDLPTNR	80

Characterization of proteins fractionated by 2D-PAGE and identified by both the DeCyder software (all gels, independent *t*-test, change in average ratio of >2 with a *t*-test of *p*<0.05 ) and Mass Spectrometry, ranked in order of change in average ratio. From the 11 spots up-regulated in SDL, 11 different proteins were identified. Spot No. is number of the spot picked and shown on an SDL 2D gel (Figure 3A); *t*-test and change in average ratio calculated by the DeCyder 2D Differential Analysis software when comparing the four SDL with the four ESDL 2D gels; Identified Proteins: full name of the protein identified by mass spectrometry, including isoform, as given by NCBI; NCBI Accession number and Gene ID number together with the official symbol provided by HUGO Gene Nomenclature Committee (HGNC) in brackets; Experimental (E) & Theoretical (T) Molecular mass (in kDa) and p*I*: the experimental (E) molecular mass and p*I* were determined by eye for each spot picked, whereas the theoretical (T) molecular mass and p*I* were determined using EditSeq (DNA lasergene 8) on the protein sequence of gi number identified; Number of peptide matched: total number of unique peptides matched to the protein identified; Mascot protein score: the protein score for that gi number is given. Protein score is defined as -10*Log(P), where P is the probability that the observed match is a random event. Protein scores greater than 66 (>66) are considered significant identifications (p<0.05). Where MSMS was performed, the calculated precursor ion mass and resulting peptide sequences are shown together with the corresponding ion score. The list of all the peptides identified for each spot is given in Table S1.

#### Proteins identified with a change in average intensity ratio of > +/−1.3

When the cut-off value of change in average intensity ratio was lowered to > +1.3 (*t*-test of *p*<0.05) an additional 19 spots exhibited an increased abundance in SDL. Of these, 14 were picked from the gels (the 5 other spots were deemed too faint to pick) and 11 were identified by mass spectrometry (Table 2 and Table S2). These include splicing factors, actin and actin binding protein cofilin-1, lamin A/C (cleaved), peptidyl-propyl *cis trans* isomerase FKBP4 and carbonic anhydrase. Western blot analysis confirmed proteolysis of lamin A/C during Epo withdrawal (Figure 4). 13 spots were found to be up-regulated in ESDL with a change in average ratio below <-1.3 and a *t*-test of *p*<0.05. Of these, 6 were picked and identified by mass spectrometry, including BTF3, a SUMO activating enzyme, RPSA (full length), polypyrimidine tract binding protein1 and Hsp105 (Table S3).

**Table 2:** Proteins with altered abundance in SDL (all gels, change in average ratio between 1.3 and 2, with a *t*-test of *p*<0.05).

Spot No.	t-test	Average ratio	Identified Proteins	Accesion number (Gene ID)	Molecular mass (kD)		p*I*		No of peptides matched	Mascot Protein Score (>66)	Precursor ion mass	MSMS Peptide sequence	Ion score
					E	T	E	T					
12	0.0083	1.74	splicing factor 3A 677ptsubunit 1 isoform 1	gi|5032087 (SF3A1, 10291)	∼75	88.9	∼4.75	5.1	14	109	905.5203	LTAQFVAR	15
											1930.8871	FNFLNPNDPYHAYYR	21
13	0.0055	1.71	Actin, beta	gi|4501885 (ACTB, 60)	∼42	42	∼5.6	5.3	10	312	1198.7054	AVFPSIVGRPR	41
											1516.7026	QEYDESGPSIVHR	67
											1790.8918	SYELPDGQVITIGNER	131
13	0.0055	1.71	Actin, gamma1	gi|4501887 (ACTG1, 71)	∼42	42	∼5.6	5.3	10	312	1198.7054	AVFPSIVGRPR	41
											1516.7026	QEYDESGPSIVHR	67
											1790.8918	SYELPDGQVITIGNER	131
14	0.013	1.71	lamin-A/C isoform 2	gi|5031875 (LMNA, 4000)	∼25	65	∼5.75	6.7	28	561	849.4828	LAVYIDR	34
											1023.5105	NIYSEELR	48
											1089.5535	SLETENAGLR	50
											1182.6113	TLEGELHDLR	73
											1629.8079	LQEKEDLQELNDR	112
15	0.018	1.65	peptidyl-prolyl cis-trans isomerase FKBP4	gi|4503729 (FKBP4, 2288)	∼50	51.8	∼5.25	5.3	20	313	1697.8717	RGEAHLAVNDFELAR	56
											1950.9443	FEIGEGENLDLPYGLER	97
16	0.0016	1.62	heterogeneous nuclear 677ptribonu-cleoproteins C1/C2 isoform b	gi|117190174 (HNRPC, 3183)	∼36	32.3	∼5.2	4.8	9	218	943.5723	VPPPPPIAR	56
											1329.6586	GFAFVQYVNER	85
16	0.0016	1.62	transformer-2 protein 677pthomolog beta	gi|4759098 (TRA2B, 6434)	∼36	33.7	∼5.2	11.3	6	133	1810.897	YGPIADVSIVYDQQSR	91
17	0.0092	1.6	protein CutA isoform 2	gi|7706244 (CUTA, 51596)	∼14	16.8	∼4.5	5.1	2	85	1533.8271	TQSSLVPALTDFVR	74
17	0.0092	1.6	U6 snRNA-associated 677ptSm-like protein LSm3	gi|7657315 (LSM3, 27258)	∼14	11.8	∼4.5	4.5	3	83	1005.5549	NIPMLFVR	13
											1192.7048	GDGVVLVAPPLR	48
18	0.021	1.43	cofilin-1	gi|5031635 (CFL1, 1072)	∼15	18.5	5.8	8	12	296	1309.682	AVLFCLSEDKK	15
											1337.626	YALYDATYETK	43
											1790.8126	HELQANCYEEVKDR	76
											2166.0964	EILVGDVGQTVDDPYATF	41
19	0.015	1.42	cofilin-1	gi|5031635 (CFL1, 1072)	∼15	18.5	∼5.65	8.1	10	294	1309.682	AVLFCLSEDKK	45
											1337.626	YALYDATYETK	59
											1790.8126	HELQANCYEEVKDR	81
20	0.014	1.36	peptidyl-prolyl cis-trans isomerase FKBP4	gi|4503729 (FKBP4, 2288)	∼52	51.8	∼5.3	5.3	21	455	1059.4928	LYANMFER	1
											1103.5626	TQLAVCQQR	37
											1697.8717	RGEAHLAVNDFELAR	98
											1950.9443	FEIGEGENLDLPYGLER	143
21	0.0063	1.33	actin, beta	gi|4501885 (ACTB, 60)	∼40	42	∼5.4	5.3	13	187	1132.527	GYSFTTTAER	2
											1198.7054	AVFPSIVGRPR	10
											1516.7026	QEYDESGPSIVHR	29
											1790.8918	SYELPDGQVITIGNER	51
22	0.0068	1.33	carbonic anhydrase 1	gi|4502517 (CA1, 759)	∼75	28.9	∼6.5	6.9	8	258	1580.7915	ESISVSSEQLAQFR	105
											2256.0427	EIINVGHSFHVNFEDNDNR	97
22	0.0068	1.33	GMP synthase	gi|4504035 (GMPS, 8833)	∼75	76.7	∼6.5	6.7	17	160	1118.6608	VVYIFGPPVK	20
											1161.6527	HPFPGPGLAIR	41
22	0.0068	1.33	Phosphoenolpyru-vate carboxykinase	gi|66346721 (PCK2, 5106)	∼75	70.7	∼6.5	7.6	18	126	1118.5894	IFHVNWFR	17

Characterization of proteins fractionated by 2D-PAGE and identified by both the DeCyder software (all gels, independent t-test, change in average ratio between 1.3 and 2, with a *t*-test of *p*<0.05 ) and Mass Spectrometry (MS), ranked in order of change in average ratio. From the 11 spots picked, 13 different proteins were identified by MS. A full list of all the peptides identified for each spot is given in Table S2.

By monitoring the intensity of each spot across the 4 independent 2D DIGE experiments, we observed that spots from one sample pair (SDL and ESDL) were consistently outliers (see Figure 3C for an example of the Hsp90 alpha result). After exclusion of one sample pair, an additional 10 spots were found to be up-regulated in SDL with a change in average ratio above >1.3 and a *t*-test of *p*<0.05. Of these, 7 were picked and identified by mass spectrometry (Table 4 and Table S4) including several enzymes, ubiquitin-conjugating E2 enzymes, and the serine/threonine protein phosphatase PP1-alpha catalytic subunit. Furthermore, after exclusion of this sample pair, an extra 20 spots had increased abundance in ESDL with a change in average ratio below <-1.3 and a *t*-test of *p*<0.05. Of these, 18 spots were picked from the gels, of which 14 were identified by mass spectrometry (Table 5 and Table S5) including secretory pathway proteins (clathrin and dynactin), Hsp70, the Hsp90 co-chaperone p23, lamin A/C (full length), splicing and ribonuclear proteins, and the serine/threonine protein kinase PAK2.

**Table 3:** Proteins with altered abundance in ESDL (all gels, change in average ratio below <-1.3, with a *t*-test of *p*<0.05).

Spot No.	t-test	Average ratio	Identified Proteins	Accesion number (Gene ID)	Molecular mass (kD)		p*I*		No of peptides matched	Mascot Protein Score (>66)	Precursor ion mass	MSMS Peptide sequence	Ion score
					E	T	E	T					
30	0.0052	-1.42	transcription factor BTF3 isoform A	gi|83641885 (BTF3, 689)	∼17	22.2	∼7	9.4	10	434	863.5349	LQFSLKK	22
											1960.0134	VQASLAANTFTITGHAETK	144
											2416.2751	QLTEMLPSILNQLGADSLTSLR	98
											3102.5312	LGVNNISGIEEVNMFTNQGTVIHFNNPK	39
31	0.013	-1.37	transcription factor BTF3 isoform A	gi|83641885 (BTF3, 689)	∼17	22.2	∼6.4	9.4	7	233	863.5349	LQFSLKK	14
											1960.0134	VQASLAANTFTITGHAETK	145
32	0.0023	-1.35	SUMO-activating enzyme subunit 1 isoform a	gi|4885585 (SAE1, 10055)	∼37	38	∼5.2	5.1	13	113	2214.1289	NDVLDSLGISPDLLPEDFVR	16
33	0.017	-1.35	40S ribosomal protein SA	gi|9845502 (RPSA, 3921)	∼40	33	∼4.6	4.7	16	693	912.5513	LLVVTDPR	41
											1203.6481	FAAATGATPIAGR	120
											1698.8599	FTPGTFTNQIQAAFR	95
											1740.949	AIVAIENPADVSVISSR	172
											2081.0562	FTPGTFTNQIQAAFREPR	112
34	0.046	-1.34	polypyrimidine tract-binding protein 1 isoform c	gi|14165466 (PTBP1, 5725)	∼50	57	∼9	9.2	13	536	991.5432	HQNVQLPR	54
											1058.5013	DYGNSPLHR	57
											1431.7379	GQPIYIQFSNHK	102
											1897.8895	LSLDGQNIYNACCTLR	92
											2275.2769	IAIPGLAGAGNSVLLVSNLNPER	137
35	0.042	-1.31	heat shock protein 105 kDa	gi|42544159 (HSPH1, 10808)	∼100	97	∼5.25	5.2	23	267	1418.6586	NAVEEYVYEFR	60
											1479.7074	AGGIETIANEFSDR	67

Characterization of proteins fractionated by 2D-PAGE and identified by both the DeCyder software (all gels, independent *t*-test, change in average ratio below <-1.3, with a *t*-test of *p*<0.05 ) and Mass Spectrometry (MS), ranked in order of change in average ratio. From the 6 spots picked, 5 different proteins were identified by MS. A full list of all the peptides identified for each spot is given in Table S3.

**Table 4:** Proteins with altered abundance in SDL (3 gels, independent t-test, change in average ratio above >1.3, with a *t*-test of *p*<0.05).

Spot No.	t-test	Average ratio	Identified Proteins	Accesion number (Gene ID)	Molecular mass (kD)		p*I*		No of peptides matched	Mascot Protein Score (>66)	Precursor ion mass	MSMS Peptide sequence	Ion score
					E	T	E	T					
23	0.019	1.88	flavin reductase	gi|4502419 (BLVRB, 645)	∼15	22.1	∼7	7.5	6	256	1167.6117	LQAVTDDHIR	62
											1493.6901	NDLSPTTVMSEGAR	8
											1512.8744	TVAGQDAVIVLLGTR	117
											2469.3096	LPSEGPRPAHVVVGDVLQAADVDK	12
24	0.029	1.76	ubiquitin-conju-gating enzyme E2	gi|40806164 (UBE2V1, 7335)	∼15	19.3	∼7.3	8.6	13	225	856.525	VVLQELR	21
											1075.5571	YPEAPPFVR	54
											1143.5979	WTGMIIGPPR	17
24	0.029	1.76	flavin reductase	gi|4502419 (BLVRB, 645)	∼15	22.1	∼7.3	7.5	5	195	1167.6117	LQAVTDDHIR	43
											1512.8744	TVAGQDAVIVLLGTR	119
25	0.00077	1.73	serine/threonine-protein phos-phatase PP1-alpha catalytic subunit	gi|4506003 (PPP1CA, 5499)	∼35	37.5	∼5.75	6.1	19	396	1439.8046	IKYPENFFLLR	81
											1722.8268	ICGDIHGQYYDLLR	103
											1913.998	YGQFSGLNPGGRPITPPR	41
26	0.0068	1.41	enoyl-CoA hydratase, mitochondrial precursor	gi|194097323 (ECHS1, 1892)	∼25	31.4	∼5.6	8.1	8	287	1163.5957	HWDHLTQVK	17
											1466.8438	NNTVGLIQLNRPK	66
											2125.1399	AQFAQPEILIGTIPGAGGTQR	148
27	0.047	1.36	cytochrome c oxidase subunit 4	gi|4502981 (COX4I1, 1327)	<15	19.6	∼9	9.5	11	104	-	-	-
28	0.00082	1.31	haloacid dehalogenase-like hydrolase domain containing 3	gi|13654294 (HDHD3, 81932)	∼25	28	∼6	6.5	11	372	876.4937	IFQEALR	40
											1355.743	LRHPLGEAYATK	41
											1739.8711	AHGLEVEPSALEQGFR	133
											1958.9468	AQSHSFPNYGLSHGLTSR	70
29	0.046	1.31	ubiquitin-conju-gating enzyme E2 N	gi|4507793 (UBE2N, 7334)	∼13	17.1	∼5.75	6.4	13	368	970.5468	WSPALQIR	25
											1036.6401	LLAEPVPGIK	66
											1203.5964	TNEAQAIETAR	63
											1213.6688	DKWSPALQIR	62

Characterization of proteins fractionated by 2D-PAGE and identified by both the DeCyder software (3 gels, independent t-test, change in average ratio above >1.3, with a *t*-test of *p*<0.05) and Mass Spectrometry (MS), ranked in order of change in average ratio. From the 7 spots picked, 7 different proteins were identified by MS. A full list of all the peptides identified for each spot is given in Table S4.

**Table 5:** Proteins with altered abundance in ESDL (3 gels, independent t-test, change in average ratio below <-1.3, with a t-test of p<0.05).

Spot No.	t-test	Average ratio	Identified Proteins	Accesion number (Gene ID)	Molecular mass (kD)		p*I*		No of peptides matched	Mascot Protein Score (>66)	Precursor ion mass	MSMS Peptide sequence	Ion score
					E	T	E	T					
36	0.024	-1.71	clathrin light chain A	gi|4502899 (CLTA, 1211)	∼30	23.7	∼4.3	4.3	9	189	1095.5105	ELEEWYAR	28
											1407.7267	AIKELEEWYAR	17
											2352.0261	AAEEAFVNDIDESSPGTEWER	74
37	0.038	-1.49	dynactin subunit 2	gi|5453629 (DCTN2, 10540)	∼48	45	∼5.2	5	19	210	1598.8748	VSALDLAVLDQVEAR	43
											1764.8398	ENLATVEGNFASIDER	9
38	0.00305	-1.41	heat shock 70 kDa protein 4	gi|38327039 (HSPA4, 3308)	∼100	94	∼5.1	5	29	269	1321.7111	VLATAFDTTLGGR	33
											1495.7023	AGGIETIANEYSDR	5
											1735.9265	EFSITDVVPYPISLR	15
39	0.004	-1.41	polypyrimidine tract-binding protein 1	gi|14165466 (PTBP1, 5725)	∼50	57	∼9	9.2	21	337	991.5432	HQNVQLPR	20
											1431.7379	GQPIYIQFSNHK	34
											2039.0959	VTPQSLFILFGVYGDVQR	27
											2243.1204	NNQFQALLQYADPVSAQHAK	42
											2275.2769	IAIPGLAGAGNSVLLVSNLNPER	35
40	0.0001	-1.39	eukaryotic translation initiation factor 4H	gi|11559923 (EIF4H, 7458)	∼27	27.4	∼5.8	7.1	10	153	957.5264	FRDGPPLR	7
											1393.6958	EALTYDGALLGDR	55
											2351.2578	TVATPLNQVANPNSAIFGGARPR	17
41	0.024	-1.38	heterogeneous nuclear ribonu-cleoprotein K	gi|14165437 (HNRNPK, 3190)	∼60	51	∼5.2	5.1	17	192	1194.6993	NLPLPPPPPPR	38
											1780.7985	TDYNASVSVPDSSGPER	32
42	0.02	-1.37	heterogeneous nuclear ribonu-cleoproteins C1/C2	gi|117190174 (HNRPC, 3183)	∼37	32.3	∼5.1	4.8	13	240	943.5723	VPPPPPIARS	26
											1316.7936	VFIGNLNTLVVK	10
											1329.6586	GFAFVQYVNER	66
											2117.8757	AAEMYGSSFDLDYDFR	34
43	0.0019	-1.36	HSP90 co-chaperone p23	gi|23308579 (PTGES3, 10728)	∼18	18.7	∼4.3	4.1	10	113	921.4644	SILCCLR	15
											1131.5542	KGESGQSWPR	4
44	0.0086	-1.35	ATP-dependent RNA helicase DDX1	gi|4826686 (DDX1, 1653)	∼80	82.4	∼6.5	7.1	23	150	-	-	-
45	0.014	-1.34	heterogeneous nuclear ribonu-cleoproteins C1/C2	gi|117190174 (HNRPC, 3183)	∼37	32.3	∼5.1	4.8	12	276	943.5723	VPPPPPIAR	20
											1316.7936	VFIGNLNTLVVK	11
											1329.6586	GFAFVQYVNER	46
											1698.9094	MIAGQVLDINLAAEPK	38
											2117.8757	SAAEMYGSSFDLDYDFR	68
46	0.00096	-1.33	lamin-A/C	gi|5031875 (LMNA, 4000)	∼60	65	∼5.8	6.7	25	199	-	-	-
47	0.011	-1.32	heterogeneous nuclear ribonu-cleoprotein K	gi|14165437 (HNRNPK, 3190)	∼60	51	∼5.3	5.1	18	235	1053.6415	VVLIGGKPDR	1
											1194.6993	NLPLPPPPPPR	49
											1518.9365	LLIHQSLAGGIIGVK	34
											1780.7985	TDYNASVSVPDSSGPER	16
48	0.027	-1.32	heterogeneous nuclear ribonu-cleoproteins C1/C2	gi|117190174 (HNRPC, 3183)	∼37	32.3	∼5.1	4.8	12	223	943.5723	VPPPPPIAR	19
											1316.7936	VFIGNLNTLVVK	15
											1329.6586	GFAFVQYVNER	50
											1698.9094	MIAGQVLDINLAAEPK	20
											2117.8757	SAAEMYGSSFDLDYDFR	27
49	0.027	-1.32	alanyl-tRNA synthetase	gi|109148542 (AARS, 16)	∼110	107	∼5.25	5.3	21	155	1408.6743	AVFDETYPDPVR	33
											1573.8009	VGDQVWLFIDEPR	5
											1605.8635	GGYVLHIGTIYGDLK	3
50	0.032	-1.32	Heterogeneous nuclear ribonu-cleoprotein K	gi|14165437 (HNRNPK, 3190)	∼60	51	∼5.3	5.1	16	209	1194.6993	NLPLPPPPPPR	50
											1518.9365	LLIHQSLAGGIIGVK	28
											1780.7985	TDYNASVSVPDSSGPER	19
51	0.0054	-1.31	acylamino-acid-releasing enzyme	gi|23510451 (APEH, 327)	∼80	81.2	∼5.2	5.3	12	81	1688.8854	QVLLSEPEEAAALYR	30
52	0.0095	-1.31	serine/threonine-protein kinase PAK 2	gi|32483399 (PAK2, 5062)	∼55	58	∼5.6	5.8	14	102	2078.0601	ECLQALEFLHANQVIHR	15
53	0.0053	-1.3	elongation factor 1-beta	gi|4503477 (EEF1B2, 1933)	∼28	24.8	∼4.5	4.3	8	108	945.5767	LVPVGYGIK	6
											1603.8326	SPAGLQVLNDYLADK	35
											3445.6733	SYIEGYVPSQADVAVFEAVSSPPPADLCHALR	7

Characterization of proteins fractionated by 2D-PAGE and identified by both the DeCyder software (3 gels, independent *t*-test, change in average ratio below <-1.3, with a *t*-test of *p*<0.05 ) and Mass Spectrometry (MS), ranked in order of change in average ratio. From the 18 spots picked, 14 different proteins were identified by MS. A full list of all the peptides identified for each spot is given in Table S5.

**Figure 5:**
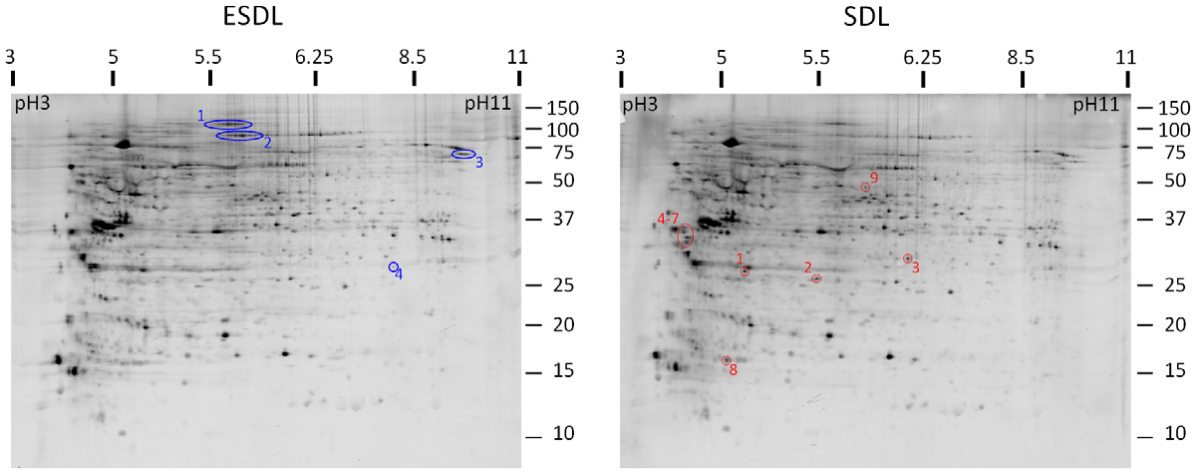
ESDL and SDL 2D gels stained for phosphoproteins. 2D gels of total cell lysates pooled from the 4 experiments harvested in 12 hour ESDL (+Epo) or SDL (no Epo) were stained with Pro-Q Diamond to detect phosphoproteins. Changes in protein phosphorylation between the two culture conditions were identified using Image Quant v5.2 software analysis and the corresponding spots (4 spots in ESDL circled in blue and 9 spots in SDL circled in red), were picked and analysed by mass spectrometry and are listed in Table 6 and Table 7.

Investigating the changes in protein phosphorylation within the proteome after Epo withdrawal revealed 13 phospho-protein changes between ESDL and SDL (Figure 5). Of these 13 spots, 9 had increased phosphorylation in SDL compared to ESDL and these were identified by mass spectrometry (Table 6 and Table S6). The proteins detected as having potentially increased phosphorylation in SDL conditions included nascent polypeptide associated complex alpha subunit (NACA), Hsp27, Hsp90 alpha and beta, and lamin A/C. The other 4 spots with altered phosphorylation profiles were increased in ESDL and from these 5 proteins were identified by mass spectrometry (Table 7 and Table S7) including matrin-3, nucleolin, splicing factor 1 and an initiation factor.

**Table 6:** Phosphorylated spots up-regulated in SDL.

Spot No.	Identified Proteins	Accesion number(Gene ID)	Molecular mass (kD)		*pI*		No of Peptides matched	Mascot Protein Score (>66)	Precursor ion mass	MSMS Peptide sequence	Ion score
			E	T	E	T					
1	Nascent poly-peptide-associated complex alpha subunit isoform b	gi|5031931 (NACA, 4666)	∼25	23.4	∼5.1	4.4	5	111	1484.7267	SPASDTYIVFGEAK	19
									1549.8988	NILFVITKPDVYK	58
									1614.8334	IEDLSQQAQLAAAEK	4
2	NDUFS3 NADH dehydrogenase (ubi-quinone) Fe-S protein 3	gi|4758788 (NDUFS3, 4722)	∼25	30.2	∼5.5	7.4	18	237	1295.663	DFPLSGYVELR	16
									1366.7729	FEIVYNLLSLR	26
									1486.79	VVAEPVELAQEFR	21
2	HSPB1 heat shock 27 kDa protein 1	gi|4504517 (HSPB1, 3315)	∼25	22.8	∼5.5	6.2	9	146	1163.6207	LFDQAFGLPR	38
									1905.9916	LATQSNEITIPVTFESR	37
2	Lamin A/C	gi|5031875	∼25	65	∼5.5	6.7	16	93	-	-	-
		gi|27436948		71		8.4					
		gi|27436946 (LMNA, 4000)		74		6.8					
3	purine nucleoside phosphorylase	gi|157168362 (PNP, 4860)	∼29 KD	32	∼6.2	6.7	16	253	1022.5781	LVFGFLNGR	34
									1208.6344	FEVGDIMLIR	14
									1664.8503	DHINLPGFSGQNPLR	30
									2009.0127	LTQAQIFDYGEIPNFPR	27
4	heat shock protein HSP 90-alpha	gi|153792590 (HSP90AA1, 3320)	∼36 kD	98	∼4.3	5	15	94	1589.8759	ELHINLIPNKQDR	17
									1778.9475	HSQFIGYPITLFVEK	5
									2015.0443	VILHLKEDQTEYLEER	4
5	heat shock protein HSP 90-alpha	gi|153792590 (HSP90AA1, 3320)	∼34 kD	98	∼4.3	5	12	103	1589.8759	ELHINLIPNKQDR	28
									1778.9475	HSQFIGYPITLFVEK	17
									2015.0443	VILHLKEDQTEYLEER	14
6	heat shock protein HSP 90-beta	gi|20149594 (HSP90AB1, 3326)	∼32 kD	83	∼4.3	4.9	16	111	1194.6477	IDIIPNPQER	18
									1564.8694	ELKIDIIPNPQER	3
									1808.9581	HSQFIGYPITLYLEK	1
									2015.0443	VILHLKEDQTEYLEER	4
7	heat shock protein HSP 90-beta	gi|20149594 (HSP90AB1, 3326)	∼30 kD	83	∼4.3	4.9	14	133	1194.6477	IDIIPNPQER	7
									1311.5699	EDQTEYLEER	19
									1808.9581	HSQFIGYPITLYLEK	23
									2015.0443	VILHLKEDQTEYLEER	16
8	acidic leucine-rich nuclear phosphoprotein 32 family member B	gi|5454088 (ANP32B, 10541)	∼15kD	28.8	∼5	3.8	10	92	1566.8163	LLPQLTYLDGYDR	12
									1972.9069	SLDLFNCEVTNLNDYR	6
9	60S acidic ribosomal protein P0	gi|4506667 (RPLP0, 6175)	∼35 kD	34.3	∼5.6	5.8	12	104	1313.71	TSFFQALGITTK	16

Characterization of proteins fractionated by 2D-PAGE, stained with Pro-Q Diamond phosphoprotein stain and identified by Image Quant v5.2 software analysis as being hyper-phosphorylated in SDL and then identified by Mass Spectrometry. From the 9 spots picked, 9 different proteins were identified by MS. A full list of all the peptides identified for each spot is given in Table S6.

**Table 7:** Phosphorylated spots up-regulated in ESDL.

Spot No.	Identified Proteins	Accesion number (Gene ID)	Molecular mass (kD)		p*I*		No of Peptides matched	Mascot Protein Score (>66)	Precursor ion mass	MSMS Peptide sequence	Ion score
			E	T	E	T					
1	Matrin-3 isoform a	gi|21626466 (MATR3, 9782)	∼125	95	∼5.6	6.1	25	166	1324.6716	GNLGAGNGNLQGPR	2
2	Nucleolin	gi|55956788 (NCL, 4691)	∼100	77	∼5.65	4.5	21	189	1160.5834	SISLYYTGEK	2
									1178.5687	EVFEDAAEIR	25
									1561.6805	GFGFVDFNSEEDAK	20
									1594.7423	GYAFIEFASFEDAK	5
3	Splicing Factor 1	gi|2463198* (gi|42544130; gi|42544125; gi|42544123; gi|295842307) (SF1, 7536)	∼70	33.4 (59.7–68.6)	∼9	9.1(9–9.5)	9	71	1561.8584	AYIVQLQIEDLTR	11
4	Eukaryotic translation initiation factor 4E type 2	gi|4757702 (EIF4E2, 9470)	∼27	28.4	∼8.3	8.9	9	68	1568.7855	QIGTFASVEQFWR	2
4	Lysophospholipase- like 1	gi|20270341 (LYPLAL1, 127018)	∼27	26.3	∼8.3	7.8	9	68	1150.564	GGISNVWFDR	1
									1938.978	HSASLIFLHGSGDSGQGLR	1

Characterization of proteins fractionated by 2D-PAGE, stained with Pro-Q Diamond phosphoprotein stain and identified by Image Quant v5.2 software analysis as being hyper-phosphorylated in ESDL and then identified by Mass Spectrometry. From the 4 spots picked, 5 different proteins were identified by MS. A full list of all the peptides identified for each spot is given in Table S7.

To confirm phosphorylation and identify possible phospho-sites on Hsp90 alpha and beta proteins, and nascent polypeptide associated complex alpha protein after Epo withdrawal, 2 additional independent 12 hour SDL samples were run on separate preparative 2D gels. Spots corresponding to NACA (spot 1), Hsp90 alpha (spot 4) and Hsp90 beta (spot 6) were picked, pooled, digested and analysed by Nano LC mass spectrometry. This technique confirmed the phosphorylated status of these proteins and we were able to identify several known and novel phospho-peptides (Table 8). It should be noted that for some peptides there was more than one possible phosphorylation site, so we have included all possibilities and the known phosphorylation site within the peptide has been indicated in Table 8.

**Table 8:** Confirmation of protein phosphorylation during 12 hour SDL.

Protein name	Accession Number	Peptide sequence	Potential site of phosphorylation	Ion score
Heat shock protein HSP 90-alpha	P07900	ELIsNSSDALDKIR	Ser 50	35
		ELISNsSDALDKIR	Ser 52	51
		ELISNSsDALDKIR	Ser 53	45
				
		ADLINNLGtIAK	Thr 104	33
				
		EVsDDEAEEKEDK	Ser 231	39
		DKEVsDDEAEEK	Ser 231	53
				
		EsEDKPEIEDVGSDEEEEK	Ser 252	30
		EsEDKPEIEDVGSDEEEEKK	Ser 252	30
		EEKEsEDKPEIEDVGSDEEEEK	Ser 252	36
		ESEDKPEIEDVGsDEEEEK	Ser 263*	49
		EEKESEDKPEIEDVGsDEEEEK	Ser 263*	44
				
Heat shock protein HSP 90-beta	PO8238	ADLINNLGtIAK	Thr 104	33
				
		EKEIsDDEAEEEK	Ser 226	56
		EIsDDEAEEEKGEK	Ser 226*	28
				
Nascent polypeptide-associated complex subunit alpha	Q13765	VQGEAVSNIQENtQTPTVQEESEEEEVDETGVEVK	Thr 157	24
		VQGEAVSNIQENTQtPTVQEESEEEEVDETGVEVK	Thr 159	32
		VQGEAVSNIQENTQTPtVQEESEEEEVDETGVEVK	Thr 161*	38
		VQGEAVSNIQENTQTPTVQEEsEEEEVDETGVEVK	Ser 166	52
		VQGEAVSNIQENTQTPTVQEESEEEEVDEtGVEVK	Thr 174	23

12 hour SDL samples were fractionated by 2D PAGE and then hyper-phosphorylated spots previously identified by mass spectrometry as HSP90 alpha, Hsp90 beta and nascent polypeptide associated complex alpha subunit were subjected to Nano LC mass spectrometry to detect phosphopeptides as outlined in the materials and methods. It should be noted that for some peptides there may be more than one possible phosphorylation site, so we have included all possibilities and known phosphorylation sites within the peptide has been indicated with an *. For some peptides there are more than one possible phosphorylation site and so all matched options are presented. *Indicates a known reported phosphorylation site [43], [61].

## Discussion

In this study we sought to determine the global proteome alterations that occur in erythroblasts during Epo withdrawal. We have shown that Fas-L independent cell death occurs in immature erythroblasts during Epo withdrawal and observed activation of both caspase 8 and caspase 9, alongside other classical features of the “intrinsic” apoptosis pathway. Caspase activation and apoptosis proceed through one of two major pathways, namely the ‘extrinsic’ pathway triggered by death receptor ligation and activation of the initiator caspase 8, or the ‘intrinsic’ pathway characterised by mitochondrial outer membrane permeabilisation (MOMP), cytochrome *c* release into the cytosol and activation of the initiator caspase 9 [27]. Both pathways then converge on activating executioner caspases, such as caspase 3 [27]. There is evidence that crosstalk can occur, as caspase 8 activation leads to cytochrome c release into the cytosol via tBid [28]. Conversely, caspase 8 activation downstream of caspase 9 has been reported [29]. Growth factor withdrawal from haematopoietic cells is generally thought to result in the activation of the mitochondrial pathway [30], [31] and our studies are supportive of this. Further studies are required to determine the exact sequence of caspase activation, to specifically delineate which apoptotic pathway (extrinsic or intrinsic) is activated first and whether caspase 8 is activated downstream of caspase 9 or by a death receptor ligand (other than Fas-L) which is yet to be identified.

We used a 2D DIGE proteomic approach to provide a snapshot of the key differences in the proteomes of erythroblasts under continual expansion and erythroblasts undergoing apoptosis due to Epo deprivation. Overall we observed more alterations in protein abundance in apoptotic cells, probably because in this state cellular proteins are undergoing proteolytic cleavage by caspases or putative unknown proteases, and the resulting shift in size makes these proteins more evident in apoptotic cells. Indeed proteins known to be caspase targets were highly represented in our analysis (59% of the proteins identified (i.e. 34 proteins out of 57 in total, Tables 1–7) are known to be cleaved by caspases [32]). It is possible that proteolysis is not solely caspase-mediated but also due to cleavage by other types of proteases activated during apoptosis. However, caspases are the main proteolytic enzymes activated during apoptosis [33] and we confirmed activation of caspases in our culture system upon Epo withdrawal (Figure 3). Furthermore, the molecular weights of the proteolytic fragments detected in the lysates of apoptotic cells (Figure 4) match the theoretical molecular weight of the protein fragments that would be generated by cleavage at the mapped caspase cleavage site. For example, the caspase cleavage site identified in Hsp90 beta [34] is conserved in both Hsp90 isoforms. Western blotting conducted on apoptotic cell lysates detected a proteolytic fragment of ∼37 kD for Hsp90 alpha (N-terminus) and of ∼50 kD (C-terminus) for Hsp90 beta (Figure 4) consistent with the sizes predicted for caspase cleavage. For 14-3-3 beta, gamma and epsilon, the molecular weight of the cleaved form was only marginally smaller than that of the full length proteins, mirroring what has already been described for caspase-mediated cleavage of 14-3-3 proteins [35].

It should be noted that although we have identified many cleaved proteins more abundant in apoptotic cells, we only detected the equivalent full length protein in the living cells for RPSA (spot 6 in Table 1 and spot 33 Table 4), Lamin A/C (spot 14 Table 2 and spot 46 Table 5) and Ribonucleoprotein C1/C2. Moreover, we have identified a range of known caspase substrates but candidates such as the GATA-1 [18] were not detected by our analysis. One explanation for this may be that some key changes are obscured or masked by other proteins present on the 2D gel, and it is also likely that alterations in low abundant proteins might not be detected. In addition, approximately 30% of the spots detected as altered in our experiments were not confidently identified by mass spectrometry and this may explain some omissions. Also, the kinetics of proteolysis in response to Epo withdrawal might vary from protein to protein, such that certain proteomic changes might not be detected at the 12 hour time point but could occur earlier or later.

Many of the changes in protein abundance on Epo withdrawal observed here in our 2D DIGE comparison with living cells, are consistent with the known characteristic alterations that occur during apoptosis, reflecting the universal nature of this fundamental process. Hence, the observed changes in cellular morphology that occur during apoptosis require alterations in actin and myosin cytoskeleton and nuclear lamins, whilst essential house keeping functions such as transcription, translation and the secretory pathway are targeted for destruction (reviewed by [36]). Therefore, the observed alterations in the abundance/cleavage of cytoskeletal proteins (myosin 9, actins, cofilin) and nuclear lamins (lamin A/C), β-NAC (protein translocation to the ER), transcription factors (EIF4A) and secretory pathway proteins were to be expected. However, it is highly significant that many of the novel changes in proteins reported here which were detected as more abundant upon Epo withdrawal are multifunctional proteins such as Hsp90, 14-3-3 isoforms, SET and RPSA that have undergone proteolysis (Table 1). These proteins regulate diverse cellular processes, including cell proliferation. Thus abrogating the function of these proteins through proteolysis would influence multiple signaling pathways simultaneously, blocking proliferation and ensuring a rapid execution of cell death. It is also important to note that Hsp90, SET, RPSA have all been implicated in myeloproliferative neoplasms. Hsp90 is a therapeutic target in JAK2-dependent myeloproliferative neoplasms [37], RPSA is highly expressed in Acute Myeloid Leukaemia (AML) [38] and SET expression is induced in Chronic Myelogenous Leukemia (CML) [39].

The Hsp90 proteins are chaperones to a multitude of client proteins, most of which are involved in signal transduction (i.e. Jak2, Pim-1, Akt/PKB) and inhibition of Hsp90 disrupts multiple pathways essential to cell survival [40]. Of interest, Hsp90 protects the pro-survival protein Pim-1 from proteasomal degradation [41] and we also found that Epo-withdrawal in our system leads to loss of the Pim-1 (data not shown). Caspase cleavage of Hsp90 has been reported previously [34] but never for Epo withdrawal. To our knowledge Hsp90 beta isoform cleavage during apoptosis has never been reported. Furthermore, our observation that both Hsp90 isoforms are phosphorylated upon Epo withdrawal is significant because phosphorylation is reported to negatively regulate Hsp90 client protein interactions [42]. Additional studies are required to determine the role of Hsp90 alpha and beta phosphorylation sites during Epo withdrawal and to establish whether phosphorylation is important for inducing caspase-mediated cleavage of Hsp90 or occurs post-caspase cleavage. One possibility is that Hsp90 phosphorylation couples the caspase cleavage to proteasomal degradation, similar to nascent polypeptide associated complex alpha subunit phosphorylation [43].

The 14-3-3 proteins also regulate diverse cellular processes including cell cycle progression, proliferation and apoptosis by functioning as chaperones and adaptors targeting more than 200 proteins [44]. Mechanistically, 14-3-3 proteins can contribute to suppression of apoptosis by sequestration of pro-apoptotic client proteins. For instance 14-3-3 proteins bind phosphorylated Bad, promoting cell survival [45]. In addition, 14-3-3 proteins also regulate FOXO protein localisation, which in the absence of 14-3-3 binding would migrate to the nucleus and initiate transcription of pro-apoptotic proteins [46]. Thus cleavage of multiple 14-3-3 proteins as seen here during Epo withdrawal may initiate or potentiate other pro-apoptotic pathways.

The SET and RPSA proteins are less well studied but both proteins are involved in a broad range of cellular processes and were cleaved after Epo withdrawal. SET is also known as the inhibitor of protein phosphatase 2A (I2PP2A, [47] or the myeloid leukaemia associated oncoprotein SET/TAF-1β [48]). It is a potent inhibitor of phosphatase 2A (PP2A) and interacts with several proteins involved in the regulation of cell cycle [49]. Interestingly, the abundance of the SET binding protein ribonucleoprotein A2 (spot 4, Table 1) increased in SDL but we did not confirm whether this protein was cleaved. Ribonucleoprotein A2 protein is also overexpressed in a variety of human tumours and is a potent inhibitor of phosphatase 2A [50]. RPSA is a protein of the 40S ribosomal subunit as well as a cell surface protein that binds laminin, prion proteins and viruses [51].

Apart from our novel observation here that both Hsp90 alpha and Hsp90 beta are cleaved in pro-erythroblasts deprived of Epo, we also report that several Hsp90 co-chaperones have altered abundance in living and apoptotic pro-erythroblasts. p23/PTGE23, a member of the Hsp90 chaperone complex suggested to stabilise the Hsp90-ATP form [52] is more abundant in cells maintained in the presence of Epo (spot 43, Table 5). In contrast, cells deprived of Epo exhibit an increase in full length or cleaved peptidyl-proyl *cis*–*trans* isomerase (PPIase) immunophilin FKBP4/FKBP52 (spots 15 and 20, Table 2), which is known to bind Hsp90 and Hsp70 and is important for the intracellular trafficking of the steroid hormone receptors [53]. Cells cultured in ESDL have a higher abundance of Hsp105 (HspH1) and Hsp70 (HspA4) (spot 35, Table 4 and spot 38 Table 5, respectively). Hsp70 prevents Gata-1 cleavage by caspase 3 [18] and AIF translocation to the nucleus [19]. On the other hand, phosphorylation of Hsp27 is induced in cells deprived of Epo (spot 2, Table 6). Although we did not confirm the phosphorylation or identity of the specific phosphorylation site(s) on Hsp27, it is notable that Hsp27 phosphorylation is required for its association with GATA-1 and for inducing GATA-1 degradation [54]. Taken together these results provide further evidence that chaperone proteins play an essential role in the regulation of Epo-induced survival in erythroblasts.

Commitment to apoptosis is post-translationally regulated by reversible phosphorylation of apoptotic signalling proteins. The abundance of several signalling proteins are altered between the two conditions some of which have already been mentioned above. In ESDL, the serine/threonine kinase p21 protein (Cdc42/Rac)-activated kinase 2 (PAK2, spot 52, Table 5) was up-regulated. PAK2 is cleaved by caspases during apoptosis [55] and its up-regulation in living cells in this study might result from loss of full length PAK2 by proteolysis in dying cells (although a smaller fragment was not identified in SDL). In apoptotic erythroblasts, the serine/threonine phosphatase PP1alpha (PPP1CA, spot 25, Table 4) increased in abundance. PP1alpha is known to be pro-apoptotic because it dephosphorylates the pro-apoptotic protein Bad [56].

Finally, several proteins involved in mRNA processing, translation and post-translational modifications were altered in presence or absence of Epo (see Tables 1–5). Interestingly, the heterogeneous nuclear ribonucleoprotein K (hnRNPK) which is detected as less abundant upon Epo withdrawal (spot 41, Table 5) has been shown to prevent the production of the pro-apoptotic BclXs splice isoform [57] and BclX is well documented to be an Epo-responsive protein important for the survival of erythroblasts at the later stages of erythropoiesis [58]. Ribonucleoprotein C1/C2 detected both in ESDL (Spot 42, Table 5,) and SDL (smaller Spot 16, Table 2) is reported to be induced by stimulators of apoptosis and p53, and has been proposed to regulate p53 mRNA during apoptosis [59]. Furthermore, Polypyrimidine tract binding protein 1 was increased in ESDL (spot 34 Table 4, and spot 39 Table 5) and this protein is reported to regulate apoptotic genes and susceptibility to caspase dependent apoptosis in differentiating cardiac myocytes [60]. Further work will need to be carried out to determine the roles of these proteins in survival and death of erythroblasts.

In summary, we have conducted the first ever comparison of the proteomes of expanding primary human erythroblasts and primary human erythroblasts undergoing the early phase of apoptotic death due to Epo withdrawal. This study has dramatically increased the repertoire of proteins that alter abundance during Epo withdrawal. In particular we report for the first time that several key multi-functional proteins are cleaved in response to Epo withdrawal from erythroblasts. Two of these proteins, Hsp90 alpha and Hsp90 beta, were also shown to be phosphorylated in apoptotic cells and we have identified these phosphorylation sites. This study validates the use of 2D DIGE to gain a comprehensive insight into cellular events leading to apoptosis in erythroblasts and as a means of identifying proteins whose aberrant regulation may contribute to human blood diseases. Furthermore, we provide an exciting new resource of candidate proteins, which will form the foundation for further studies on the mechanism of apoptosis caused by Epo withdrawal and also for studies on human diseases where there is ineffective erythropoiesis.

## Supporting Information

Figure S1 A) Flow cytometry analysis of cell surface markers expressed by erythroblasts between day 6 and day 10 in culture in ESDL medium. FL2 fluorescence (x axis) versus cell number (y axis) of cells labelled with the isotype control antibody (grey line) and antibodies against CD117/c-kit, CD71, GPA (BRIC256) and band3 (BRIC6). By day 9 the majority of cells are are c-kit^+^ positive, CD71^high^, GPA^low^/^med^ and band 3^low/neg^ B) Graph showing the average percentage of live erythroblasts after 24 h in ESDL or SDL, normalised to the percentage of live cells in ESDL between day 7 and day 10. This shows that similar level of cell death is achieved irrespective of the number of days expanding in culture.
(TIF)

Figure S2 **The expression of Fas and FasL was analyzed by flow cytometry for Fas and FasL expression on day 9 erythroblasts after 24 hours in the presence (ESDL) or absence (SDL) of Epo.** The figure shows that erythroblasts from both treatments express Fas. In contrast, the expression of FasL in these cells was absent and this did not change upon withdrawal of Epo.
(TIF)

Figure S3 **Erythroblasts kept in expansion medium (ESDL, +Epo, green histograms) and erythroblasts switched to SDL (no Epo, pink histograms) were analysed by flow cytometry using Annexin V, TMRE and propidium iodide at 6 hours, 12 hour and 24 hour.** After 12 hour, the cells switched to SDL start showing signs of apoptosis and this time point was chosen for proteomic analyses by 2D DIGE.
(TIF)

Table S1 **Table S1 lists all peptides identified by mass spectrometry from each individual spot detailed in** **Table 1** **.**
(DOCX)

Table S2 **Table S2 lists all peptides identified by mass spectrometry from each individual spot detailed in** **Table 2** **.**
(DOCX)

Table S3 **Table S3 lists all peptides identified by mass spectrometry from each individual spot detailed in** **Table 3** **.**
(DOCX)

Table S4 **Table S4 lists all peptides identified by mass spectrometry from each individual spot detailed in** **Table 4** **.**
(DOCX)

Table S5 **Table S5 lists all peptides identified by mass spectrometry from each individual spot detailed in** **Table 5** **.**
(DOCX)

Table S6 **Table S6 lists all peptides identified by mass spectrometry from each individual spot detailed in** **Table 6** **.**
(DOCX)

Table S7 **Table S7 lists all peptides identified by mass spectrometry from each individual spot detailed in** **Table 7** **.**
(DOCX)
