# Supplementary figures and images for: Differential Proteomic Analysis of Human Erythroblasts Undergoing Apoptosis Induced by Epo-Withdrawal
Source: PLoS One. 2012 Jun 18;7(6):e38356. doi: 10.1371/journal.pone.0038356 (PMC3377639; doi:10.1371/journal.pone.0038356)

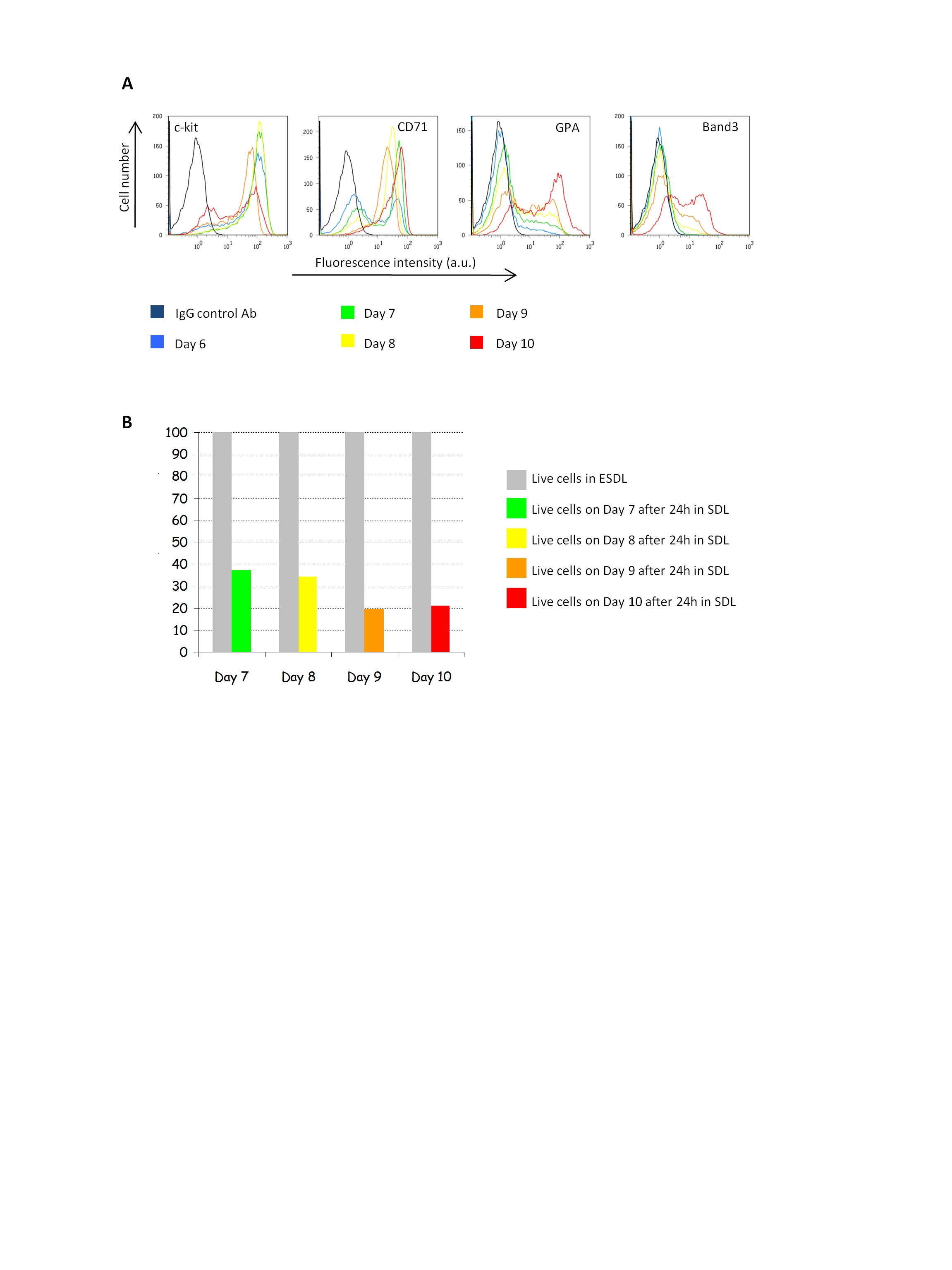

Supplement: Figure S1 — A) Flow cytometry analysis of cell surface markers expressed by erythroblasts between day 6 and day 10 in culture in ESDL medium. FL2 fluorescence (x axis) versus cell number (y axis) of cells labelled with the isotype control antibody (grey line) and antibodies against CD117/c-kit, CD71, GPA (BRIC256) and band3 (BRIC6). By day 9 the majority of cells are are c-kit+ positive, CD71high, GPAlow/med and band 3low/neg B) Graph showing the average percentage of live erythroblasts after 24 h in ESDL or SDL, normalised to the percentage of live cells in ESDL between day 7 and day 10. This shows that similar level of cell death is achieved irrespective of the number of days expanding in culture. (TIF) [file pone.0038356.s001.tif]

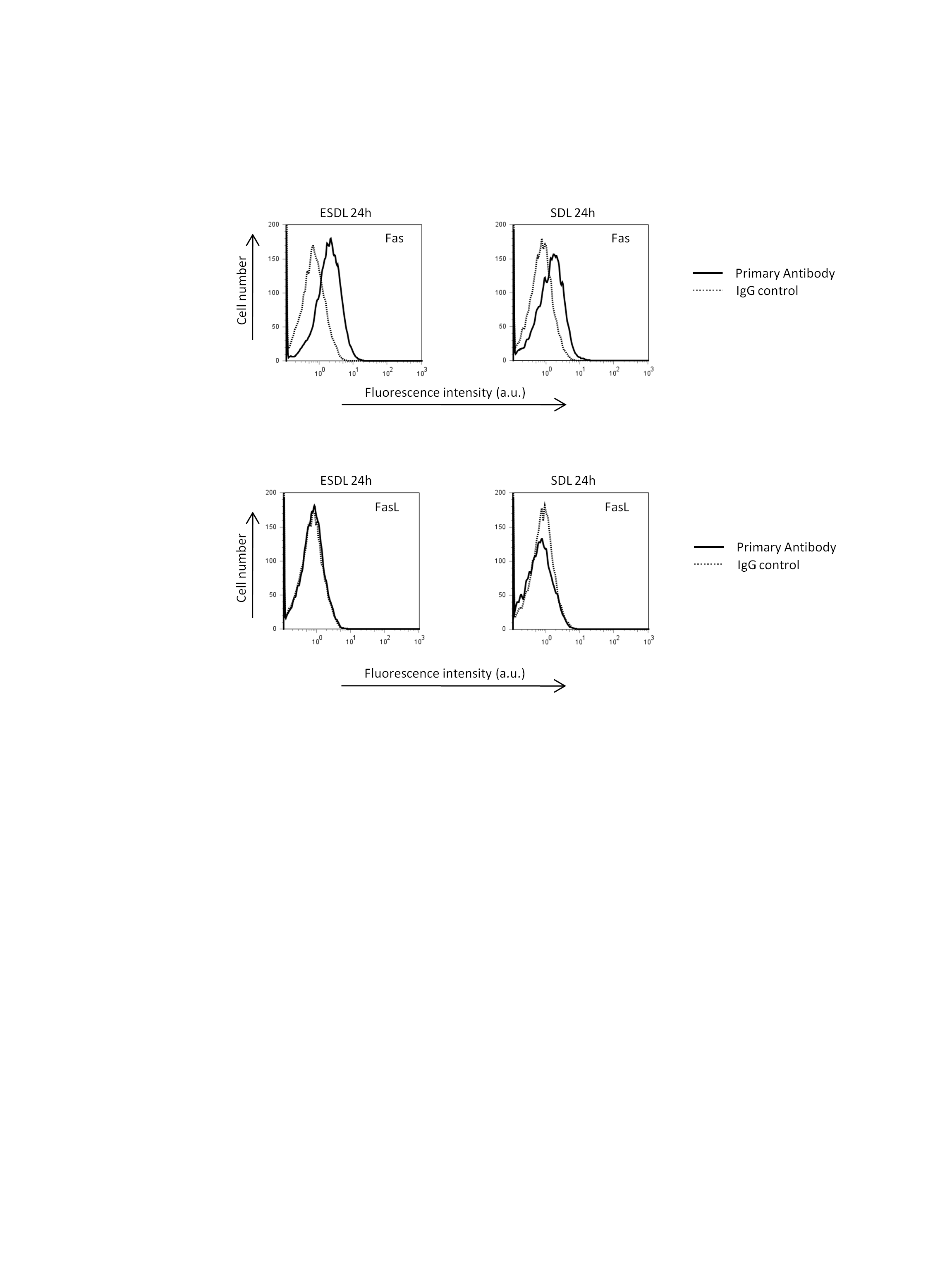

Supplement: Figure S2 — The expression of Fas and FasL was analyzed by flow cytometry for Fas and FasL expression on day 9 erythroblasts after 24 hours in the presence (ESDL) or absence (SDL) of Epo. The figure shows that erythroblasts from both treatments express Fas. In contrast, the expression of FasL in these cells was absent and this did not change upon withdrawal of Epo. (TIF) [file pone.0038356.s002.tif]

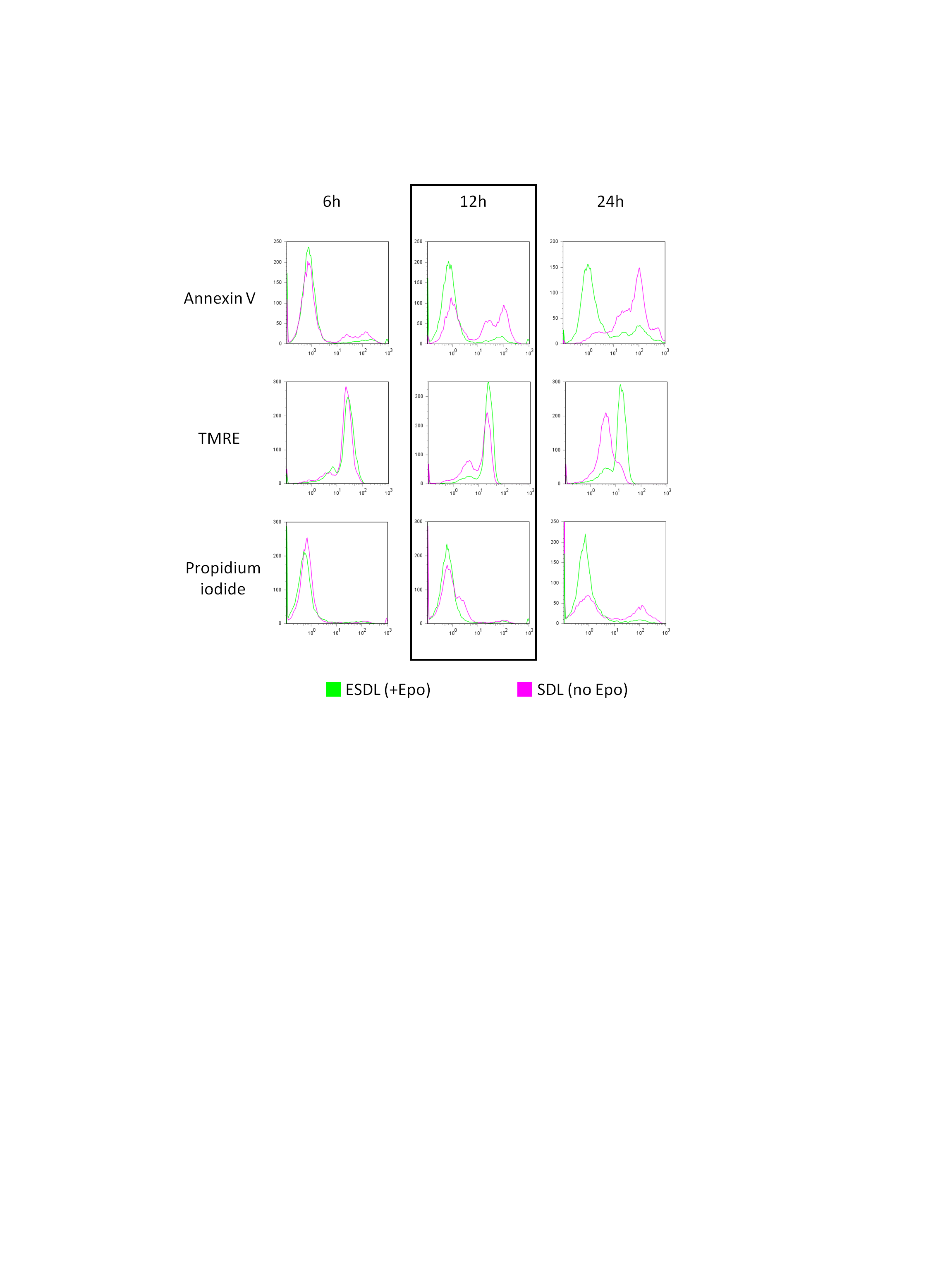

Supplement: Figure S3 — Erythroblasts kept in expansion medium (ESDL, +Epo, green histograms) and erythroblasts switched to SDL (no Epo, pink histograms) were analysed by flow cytometry using Annexin V, TMRE and propidium iodide at 6 hours, 12 hour and 24 hour. After 12 hour, the cells switched to SDL start showing signs of apoptosis and this time point was chosen for proteomic analyses by 2D DIGE. (TIF) [file pone.0038356.s003.tif]
