# Supplementary material for: Differential Proteomic Analysis of Human Erythroblasts Undergoing Apoptosis Induced by Epo-Withdrawal
Source: PLoS One. 2012 Jun 18;7(6):e38356. doi: 10.1371/journal.pone.0038356 (PMC3377639; doi:10.1371/journal.pone.0038356)
Supplement: Table S1 — lists all peptides identified by mass spectrometry from each individual spot detailed in Table 1 . (DOCX) [file pone.0038356.s004.docx]

| **Supporting information Table S1. All peptides detected.** | | |
| --- | --- | --- |
|  | | |
| **Spot No.** | **Identified Proteins** | **Peptides detected** |
| 1 | SET isoform 2 | QPFFQKR |
|  |  | LRQPFFQK |
|  |  | SSQTQNKASR |
|  |  | VEVTEFEDIK |
|  |  | LNEQASEEILK |
|  |  | EFHLNESGDPSSK |
|  |  | LNEQASEEILKVEQK |
|  |  | IDFYFDENPYFENK |
|  |  | EQQEAIEHIDEVQNEIDR |
|  |  |  |
| 2 | 14-3-3 gamma | NLLSVAYK |
|  |  | YLAEVATGEK |
|  |  | DSTLIMQLLR |
|  |  | YLAEVATGEKR |
|  |  | EHMQPTHPIR |
|  |  | VDPEQLVQKAR |
|  |  | NVTELNEPLSNEER |
|  |  | ARLAEQAEGYDDMAAAMK |
|  |  | LAEQAEGYDDMAAAMKNVTELNEPLSNEER |
|  |  |  |
| 3 | 14-3-3 beta/alpha | LAEQAER |
|  |  | VISSIEQK |
|  |  | NLLSVAYK |
|  |  | MKGDYFR |
|  |  | EMQPTHPIR |
|  |  | YLSEVASGDNK |
|  |  | DSTLIMQLLR |
|  |  | KEMQPTHPIR |
|  |  | YLIPNATQPESK |
|  |  | AVTEQGHELSNEER |
|  |  | TAFDEAIAELDTLNEESYK |
|  |  | QTTVSNSQQAYQEAFEISK |
|  |  | QTTVSNSQQAYQEAFEISKK |
|  |  |  |
| 4 | heterogeneous nuclear ribonucleoproteins A2/B1 isoform A2 | KLFVGGIK |
|  |  | GGNFGFGDSR |
|  |  | DYFEEYGK |
|  |  | IDTIEIITDR |
|  |  | GGGGNFGPGPGSNFR |
|  |  | YHTINGHNAEVR |
|  |  | EESGKPGAHVTVKK |
|  |  | LTDCVVMRDPASK |
|  |  | YHTINGHNAEVRK |
|  |  | GFGFVTFDDHDPVDK |
|  |  | RGFGFVTFDDHDPVDK |
|  |  | LFVGGIKEDTEEHHLR |
|  |  | NMGGPYGGGNYGPGGSGGSGGYGGR |
|  |  | DYFEEYGKIDTIEIITDR |
|  |  | GFGFVTFDDHDPVDKIVLQK |
|  |  |  |
| 5 | heat shock protein HSP 90 alpha isoform 2 | DNSTMGYMAAK |
|  |  | ELHINLIPNK |
|  |  | ADLINNLGTIAK |
|  |  | RAPFDLFENR |
|  |  | EDQTEYLEER |
|  |  | TLTIVDTGIGMTK |
|  |  | ELISNSSDALDKIR |
|  |  | ELHINLIPNKQDR |
|  |  | HSQFIGYPITLFVEK |
|  |  | KHSQFIGYPITLFVEK |
|  |  | VILHLKEDQTEYLEER |
|  |  | HNDDEQYAWESSAGGSFTVR |
|  |  |  |
| 6 | 40S ribosomal protein SA | LLVVTDPR |
|  |  | SDGIYIINLK |
|  |  | FAAATGATPIAGR |
|  |  | SDGIYIINLKR |
|  |  | YVDIAIPCNNK |
|  |  | GAHSVGLMWWMLAR |
|  |  | FTPGTFTNQIQAAFR |
|  |  | DPEEIEKEEQAAAEK |
|  |  | AIVAIENPADVSVISSR |
|  |  | EHPWEVMPDLYFYR |
|  |  | FTPGTFTNQIQAAFREPR |
|  |  | AIVAIENPADVSVISSRNTGQR |
|  |  | FLAAGTHLGGTNLDFQMEQYIYK |
|  |  | FLAAGTHLGGTNLDFQMEQYIYKR |
|  |  | ADHQPLTEASYVNLPTIALCNTDSPLR |
|  |  |  |
| 7 | heat shock protein HSP 90-beta | ADHGEPIGR |
|  |  | ALLFIPRR |
|  |  | IDIIPNPQER |
|  |  | APFDLFENKK |
|  |  | ADLINNLGTIAK |
|  |  | ELISNASDALDK |
|  |  | DNSTMGYMMAK |
|  |  | EISDDEAEEEK |
|  |  | LGIHEDSTNRR |
|  |  | IRYESLTDPSK |
|  |  | EDQTEYLEER |
|  |  | TLTLVDTGIGMTK |
|  |  | YESLTDPSKLDSGK |
|  |  | ELISNASDALDKIR |
|  |  | ELKIDIIPNPQER |
|  |  | HSQFIGYPITLYLEK |
|  |  | KHSQFIGYPITLYLEK |
|  |  | VILHLKEDQTEYLEER |
|  |  | HNDDEQYAWESSAGGSFTVR |
|  |  |  |
| 8 | 14-3-3 epsilon | LAEQAER |
|  |  | NLLSVAYK |
|  |  | IISSIEQK |
|  |  | EAAENSLVAYK |
|  |  | DSTLIMQLLR |
|  |  | HLIPAANTGESK |
|  |  | YLAEFATGNDR |
|  |  | YLAEFATGNDRK |
|  |  | VAGMDVELTVEER |
|  |  | LICCDILDVLDK |
|  |  | AASDIAMTELPPTHPIR |
|  |  | AAFDDAIAELDTLSEESYK |
|  |  | LGLALNFSVFYYEILNSPDR |
|  |  |  |
| 9 | Myosin 9 | GDLPFVVPR |
|  |  | VAAYDKLEK |
|  |  | GDLPFVVPRR |
|  |  | RGDLPFVVPR |
|  |  | TEMEDLMSSK |
|  |  | DVLLQVDDER |
|  |  | ASREEILAQAK |
|  |  | GMFRTVGQLYK |
|  |  | LRLEVNLQAMK |
|  |  | ALSLARALEEAMEQK |
|  |  | NFINNPLAQADWAAKK |
|  |  | ANLQIDQINTDLNLER |
|  |  | LQQELDDLLVDLDHQR |
|  |  | TFHIFYYLLSGAGEHLK |
|  |  | SMEAEMIQLQEELAAAER |
|  |  | QAQQERDELADEIANSSGK |
|  |  | ELEDATETADAMNREVSSLK |
|  |  | IAQLEEELEEEQGNTELINDR |
|  |  | DFSALESQLQDTQELLQEENR |
|  |  |  |
| 10 | stathmin isoform a | ASGQAFELILSPR |
|  |  |  |
| 11 | eukaryotic initiation factor 4A- | ENYIHR |
|  |  | VFDMLNR |
|  |  | FMRDPIR |
|  |  | EELTLEGIR |
|  |  | VFDMLNRR |
|  |  | QFYINVER |
|  |  | VLITTDLLAR |
|  |  | ATQALVLAPTR |
|  |  | KEELTLEGIR |
|  |  | GYDVIAQAQSGTGK |
|  |  | KGVAINMVTEEDK |
|  |  | GFKDQIYDIFQK |
|  |  | MFVLDEADEMLSR |
|  |  | LQMEAPHIIVGTPGR |
|  |  | QFYINVEREEWK |
|  |  | GIYAYGFEKPSAIQQR |
|  |  | DFTVSAMHGDMDQKER |
|  |  | GIDVQQVSLVINYDLPTNR |
|  |  |  |
