# Supplementary material for: Differential Proteomic Analysis of Human Erythroblasts Undergoing Apoptosis Induced by Epo-Withdrawal
Source: PLoS One. 2012 Jun 18;7(6):e38356. doi: 10.1371/journal.pone.0038356 (PMC3377639; doi:10.1371/journal.pone.0038356)
Supplement: Table S2 — lists all peptides identified by mass spectrometry from each individual spot detailed in Table 2 . (DOCX) [file pone.0038356.s005.docx]

| **Supporting information Table S2. All peptides detected.** | | |
| --- | --- | --- |
| **Spot No.** | **Identified proteins** | **Peptides detected** |
| 12 | splicing factor 3A subunit 1 isoform 1 | MQEHMR |
|  |  | FQERER |
|  |  | LTAQFVAR |
|  |  | NGPEFEAR |
|  |  | QFLTQLMQK |
|  |  | EVLDQVCYR |
|  |  | NGPEFEARIR |
|  |  | IRQNEINNPK |
|  |  | VQAQVIQETIVPK |
|  |  | RTDIFGVEETAIGK |
|  |  | FNFLNPNDPYHAYYR |
|  |  | VMQQQQQTTQQQLPQK |
|  |  | EDSAPSKPVVGIIYPPPEVR |
|  |  | NYQFDFLRPQHSLFNYFTK |
|  |  |  |
| 13 | Actin, beta | IIAPPERK |
|  |  | AGFAGDDAPR |
|  |  | GYSFTTTAER |
|  |  | AVFPSIVGRPR |
|  |  | IWHHTFYNELR |
|  |  | QEYDESGPSIVHR |
|  |  | SYELPDGQVITIGNER |
|  |  | VAPEEHPVLLTEAPLNPK |
|  |  | DLYANTVLSGGTTMYPGIADR |
|  |  | EKLCYVALDFEQEMATAASSSSLEK |
|  |  |  |
| 13 | Actin, gamma1 | IIAPPERK |
|  |  | AGFAGDDAPR |
|  |  | GYSFTTTAER |
|  |  | AVFPSIVGRPR |
|  |  | IWHHTFYNELR |
|  |  | QEYDESGPSIVHR |
|  |  | SYELPDGQVITIGNER |
|  |  | VAPEEHPVLLTEAPLNPK |
|  |  | DLYANTVLSGGTTMYPGIADR |
|  |  | EKLCYVALDFEQEMATAASSSSLEK |
|  |  |  |
| 14 | lamin-A/C isoform 2 | VREEFK |
|  |  | LQLELSK |
|  |  | LAVYIDR |
|  |  | METPSQR |
|  |  | NIYSEELR |
|  |  | EGDLIAAQAR |
|  |  | QLQDEMLR |
|  |  | ARLQLELSK |
|  |  | LQLELSKVR |
|  |  | SLETENAGLR |
|  |  | EDLQELNDR |
|  |  | ITESEEVVSR |
|  |  | AAYEAELGDAR |
|  |  | LVEIDNGKQR |
|  |  | KQLQDEMLR |
|  |  | TLEGELHDLR |
|  |  | QLQDEMLRR |
|  |  | LKDLEALLNSK |
|  |  | EAALSTALSEKR |
|  |  | QNGDDPLLTYR |
|  |  | AAYEAELGDARK |
|  |  | RTLEGELHDLR |
|  |  | SGAQASSTPLSPTR |
|  |  | NIYSEELRETK |
|  |  | LRITESEEVVSR |
|  |  | LQTMKEELDFQK |
|  |  | LQEKEDLQELNDR |
|  |  | TLEGELHDLRGQVAK |
|  |  |  |
| 15 | peptidyl-prolyl cis-trans isomerase FKBP4 | YELHLK |
|  |  | QALLQYK |
|  |  | DKLFDQR |
|  |  | LYANMFER |
|  |  | VLQLYPNNK |
|  |  | TQLAVCQQR |
|  |  | ALELDSNNEK |
|  |  | FQIPPNAELK |
|  |  | KLYANMFER |
|  |  | EGTGTEMPMIGDR |
|  |  | LQAFSAAIESCNK |
|  |  | LASHLNLAMCHLK |
|  |  | GEAHLAVNDFELAR |
|  |  | VFVHYTGWLLDGTK |
|  |  | RGEAHLAVNDFELAR |
|  |  | AKAEASSGDHPTDTEMK |
|  |  | FEIGEGENLDLPYGLER |
|  |  | GEHSIVYLKPSYAFGSVGK |
|  |  | GEGYAKPNEGAIVEVALEGYYK |
|  |  | VGEVCHITCKPEYAYGSAGSPPK |
|  |  |  |
| 16 | Heterogeneous nuclear ribonu-cleoproteins C1/C2 isoform b | MASNVTNK |
|  |  | MYSYPAR |
|  |  | VPPPPPIAR |
|  |  | QAVEMKNDK |
|  |  | VFIGNLNTLVVK |
|  |  | SEEEQSSSSVKK |
|  |  | GFAFVQYVNER |
|  |  | MIAGQVLDINLAAEPK |
|  |  | SAAEMYGSSFDLDYDFQR |
|  |  |  |
| 16 | transformer-2 protein homolog beta | IRVDFSITK |
|  |  | SASRSGSAHGSGK |
|  |  | AAQDRDQIYR |
|  |  | GYDDRDYYSR |
|  |  | GFAFVYFENVDDAK |
|  |  | YGPIADVSIVYDQQSR |
|  |  |  |
| 17 | protein CutA isoform 2 | TQSSLVPALTDFVR |
|  |  | MPALLPVASRLLLLPR |
|  |  |  |
| 17 | U6 snRNA-associated Sm-like protein LSm3 | NIPMLFVR |
|  |  | RNIPMLFVR |
|  |  | GDGVVLVAPPLR |
|  |  |  |
| 18 | Cofilin-1 | NIILEEGK |
|  |  | VFNDMKVR |
|  |  | MLPDKDCR |
|  |  | AVLFCLSEDKK |
|  |  | YALYDATYETK |
|  |  | LGGSAVISLEGKPL |
|  |  | HELQANCYEEVK |
|  |  | HELQANCYEEVKDR |
|  |  | KEDLVFIFWAPESAPLK |
|  |  | LTGIKHELQANCYEEVK |
|  |  | EILVGDVGQTVDDPYATFVK |
|  |  | NIILEEGKEILVGDVGQTVDDPYATFVK |
|  |  |  |
| 19 | Cofilin-1 | VFNDMKVR |
|  |  | MLPDKDCR |
|  |  | AVLFCLSEDKK |
|  |  | YALYDATYETK |
|  |  | LGGSAVISLEGKPL |
|  |  | HELQANCYEEVK |
|  |  | HELQANCYEEVKDR |
|  |  | KEDLVFIFWAPESAPLK |
|  |  | EILVGDVGQTVDDPYATFVK |
|  |  | NIILEEGKEILVGDVGQTVDDPYATFVK |
|  |  |  |
| 20 | Peptidyl-prolyl cis-trans isomerase FKBP4 | YELHLK |
|  |  | QALLQYK |
|  |  | DKLFDQR |
|  |  | LYANMFER |
|  |  | VLQLYPNNK |
|  |  | TQLAVCQQR |
|  |  | FQIPPNAELK |
|  |  | KLYANMFER |
|  |  | FSFDLGKGEVIK |
|  |  | LQAFSAAIESCNK |
|  |  | LASHLNLAMCHLK |
|  |  | GEAHLAVNDFELAR |
|  |  | VFVHYTGWLLDGTK |
|  |  | RGEAHLAVNDFELAR |
|  |  | FQIPPNAELKYELHLK |
|  |  | FEIGEGENLDLPYGLER |
|  |  | GEHSIVYLKPSYAFGSVGK |
|  |  | IVSWLEYESSFSNEEAQK |
|  |  | GEGYAKPNEGAIVEVALEGYYK |
|  |  | VGEVCHITCKPEYAYGSAGSPPK |
|  |  | GEGYAKPNEGAIVEVALE |
|  |  |  |
| 21 | actin, beta | IIAPPERK |
|  |  | AGFAGDDAPRI |
|  |  | GYSFTTTAER |
|  |  | AVFPSIVGRPR |
|  |  | WHHTFYNELR |
|  |  | QEYDESGPSIVHR |
|  |  | MQKEITALAPSTMK |
|  |  | SYELPDGQVITIGNER |
|  |  | VAPEEHPVLLTEAPLNPK |
|  |  | DLYANTVLSGGTTMYPGIADR |
|  |  | KYSVWIGGSILASLSTFQQMWISK |
|  |  | EKLCYVALDFEQEMATAASSSSLEK |
|  |  | TTGIVMDSGDGVTHTVPIYEGYALPHAILR |
|  |  |  |
| 22 | carbonic anhydrase 1 | GGPFSDSYR |
|  |  | ESISVSSEQLAQFR |
|  |  | YSAELHVAHWNSAK |
|  |  | LYPIANGNNQSPVDIK |
|  |  | HDTSLKPISVSYNPATAK |
|  |  | EIINVGHSFHVNFEDNDNR |
|  |  | SLLSNVEGDNAVPMQHNNRPTQPLK |
|  |  | LFQFHFHWGSTNEHGSEHTVDGVK |
|  |  |  |
| 22 | GMP synthase | MVTEIKK |
|  |  | KPHTLLQR |
|  |  | VVYIFGPPVK |
|  |  | HPFPGPGLAIR |
|  |  | ELGLPEELVSR |
|  |  | QADFEAHNILR |
|  |  | LIPRMCHNVNR |
|  |  | DEPDWESLIFLAR |
|  |  | GLQKEEVVLLTHGDSVDK |
|  |  | EDGVFNISVDNTCSLFR |
|  |  | NFLYDIAGCSGTFTVQNR |
|  |  | ALNQEQVIAVHIDNGFMR |
|  |  | EPPTDVTPTFLTTGVLSTLR |
|  |  | ISQMPVILTPLHFDRDPLQK |
|  |  | VINAAHSFYNGTTTLPISDEDR |
|  |  | VINAAHSFYNGTTTLPISDEDRTPR |
|  |  | ELFVQSEIFPLETPAFAIKEQGFR |
|  |  |  |
| 22 | Phosphoenolpyru-vate carboxykinase | VLDWICR |
|  |  | TVIVTPSQR |
|  |  | EGALDLSGLR |
|  |  | HGVFVGSAMR |
|  |  | YNNCWLAR |
|  |  | DFWEQEVR |
|  |  | IFHVNWFR |
|  |  | TLIGHVPDQR |
|  |  | YVAAAFPSACGK |
|  |  | EVLAELEALER |
|  |  | VLSGDLGQLPTGIR |
|  |  | GVPLVYEAFNWR |
|  |  | VECVGDDIAWMR |
|  |  | GQLGNWMSPADFQR |
|  |  | LGTPVLQALGDGDFVK |
|  |  | EIISFGSGYGGNSLLGK |
|  |  | DEAGHFLWPGFGENAR |
|  |  | TMYVLPFSMGPVGSPLSR |
|  |  |  |
| **Complete peptide list Table 4** | | |
| 23 | flavin reductase | LQAVTDDHIR |
|  |  | NDLSPTTVMSEGAR |
|  |  | TVAGQDAVIVLLGTR |
|  |  | VVACTSAFLLWDPTK |
|  |  | VVACTSAFLLWDPTKVPPR |
|  |  | LPSEGPRPAHVVVGDVLQAADVDK |
|  |  |  |
| 24 | ubiquitin-conju-gating enzyme E2 | VVLQELR |
|  |  | VVLQELRR |
|  |  | WQNSYSIK |
|  |  | YPEAPPFVR |
|  |  | PGEVQASYLK |
|  |  | SQSKLSDEGR |
|  |  | WTGMIIGPPR |
|  |  | LMMSKENMK |
|  |  | LLEELEEGQK |
|  |  | IYSLKIECGPK |
|  |  | INMNGVNSSNGVVDPR |
|  |  | AISVLAKWQNSYSIK |
|  |  | GVGDGTVSWGLEDDEDMTLTR |
|  |  |  |
| 24 | flavin reductase | LQAVTDDHIR |
|  |  | NDLSPTTVMSEGAR |
|  |  | TVAGQDAVIVLLGTR |
|  |  | VVACTSAFLLWDPTK |
|  |  | LPSEGPRPAHVVVGDVLQAADVDK |
|  |  |  |
| 25 | serine/threonine-protein phos-phatase PP1-alpha catalytic subunit | LNLDSIIGR |
|  |  | HDLDLICR |
|  |  | LLEVQGSRPGK |
|  |  | IYGFYDECK |
|  |  | YPENFFLLR |
|  |  | NVQLTENEIR |
|  |  | GVSFTFGAEVVAK |
|  |  | IYGFYDECKR |
|  |  | IKYPENFFLLR |
|  |  | QSLETICLLLAYK |
|  |  | FLHKHDLDLICR |
|  |  | AHQVVEDGYEFFAK |
|  |  | ICGDIHGQYYDLLR |
|  |  | AHQVVEDGYEFFAKR |
|  |  | YGQFSGLNPGGRPITPPR |
|  |  | EIFLSQPILLELEAPLK |
|  |  | TFTDCFNCLPIAAIVDEK |
|  |  | IFCCHGGLSPDLQSMEQIR |
|  |  | LFEYGGFPPESNYLFLGDYVDR |
|  |  |  |
| 26 | enoyl-CoA hydratase, mitochondrial | HWDHLTQVK |
|  |  | LFYSTFATDDR |
|  |  | SLAMEMVLTGDR |
|  |  | LFYSTFATDDRK |
|  |  | NNTVGLIQLNRPK |
|  |  | ESVNAAFEMTLTEGSK |
|  |  | EMQNLSFQDCYSSK |
|  |  | AQFAQPEILIGTIPGAGGTQR |
|  |  |  |
| 27 | cytochrome c oxidase subunit 4 | ESFAEMNR |
|  |  | VNPIQGLASK |
|  |  | DHPLPEVAHVK |
|  |  | ASWSSLSMDEK |
|  |  | FKESFAEMNR |
|  |  | RDHPLPEVAHVK |
|  |  | SEDFSLPAYMDR |
|  |  | HYVYGPLPQSFDK |
|  |  | SEDFSLPAYMDRR |
|  |  | ASWSSLSMDEKVELYR |
|  |  | HYVYGPLPQSFDKEWVAK |
|  |  |  |
| 28 | haloacid dehalogenase-like hydrolase domain containing 3 | LLTWDVK |
|  |  | IFQEALR |
|  |  | LAVISNFDR |
|  |  | HPLGEAYATK |
|  |  | LAVISNFDRR |
|  |  | LRHPLGEAYATK |
|  |  | AHGLEVEPSALEQGFR |
|  |  | AQSHSFPNYGLSHGLTSR |
|  |  | AVGMHSFLVVGPQALDPVVR |
|  |  | DFSHPCTWQVLDGAEDTLR |
|  |  | LAHMEPVVAAHVGDNYLCDYQGPR |
|  |  |  |
| 29 | ubiquitin-conju-gating enzyme E2 | MAGLPRR |
|  |  | ICLDILK |
|  |  | WSPALQIR |
|  |  | IYHPNVDK |
|  |  | AEPDESNAR |
|  |  | LLAEPVPGIK |
|  |  | TNEAQAIETAR |
|  |  | DKWSPALQIR |
|  |  | IYHPNVDKLGR |
|  |  | TNEAQAIETARAWTR |
|  |  | LELFLPEEYPMAAPK |
|  |  | LLAEPVPGIKAEPDESNAR |
|  |  | YFHVVIAGPQDSPFEGGTFK |
|  |  |  |
