# Supplementary material for: Differential Proteomic Analysis of Human Erythroblasts Undergoing Apoptosis Induced by Epo-Withdrawal
Source: PLoS One. 2012 Jun 18;7(6):e38356. doi: 10.1371/journal.pone.0038356 (PMC3377639; doi:10.1371/journal.pone.0038356)
Supplement: Table S3 — lists all peptides identified by mass spectrometry from each individual spot detailed in Table 3 . (DOCX) [file pone.0038356.s006.docx]

| **Supporting information Table S3. All peptides detected** | | |
| --- | --- | --- |
| **Spot No.** | **Identified proteins** | **Peptides Detected** |
| 30 | Transcription factor BTF3 isoform A | LQFSLKK |
|  |  | RLAEALPK |
|  |  | GGTRGQEPQMK |
|  |  | VQASLAANTFTITGHAETK |
|  |  | QLTEMLPSILNQLGADSLTSLR |
|  |  | QLTEMLPSILNQLGADSLTSLRR |
|  |  | APLATGEDDDDEVPDLVENFDEASK |
|  |  | LGVNNISGIEEVNMFTNQGTVIHFNNPK |
|  |  | APLATGEDDDDEVPDLVENFDEASKNEAN |
|  |  | KLGVNNISGIEEVNMFTNQGTVIHFNNPK |
|  |  |  |
| 31 | Transcription factor BTF3 isoform A | LQFSLKK |
|  |  | RLAEALPK |
|  |  | GGTRGQEPQMK |
|  |  | VQASLAANTFTITGHAETK |
|  |  | QLTEMLPSILNQLGADSLTSLR |
|  |  | LGVNNISGIEEVNMFTNQGTVIHFNNPK |
|  |  | APLATGEDDDDEVPDLVENFDEASKNEAN |
|  |  |  |
| 32 | SUMO-activating enzyme subunit 1 isoform a | LWGLEAQKR |
|  |  | LDSSETTMVK |
|  |  | EALEVDWSSEK |
|  |  | VSQGVEDGPDTKR |
|  |  | TTSDYFLLQVLLK |
|  |  | DPPHNNFFFFDGMK |
|  |  | EEAGGGISEEEAAQYDR |
|  |  | DPSSDTYEEDSELLLQIR |
|  |  | VEKEEAGGGISEEEAAQYDR |
|  |  | NDVLDSLGISPDLLPEDFVR |
|  |  | GRDPSSDTYEEDSELLLQIR |
|  |  | GLTMLDHEQVTPEDPGAQFLIR |
|  |  | FFTGDVFGYHGYTFANLGEHEFVEEK |
|  |  |  |
| 33 | 40S ribosomal protein SA | LLVVTDPR |
|  |  | SDGIYIINLK |
|  |  | FAAATGATPIAGR |
|  |  | KSDGIYIINLK |
|  |  | SDGIYIINLKR |
|  |  | YVDIAIPCNNK |
|  |  | GAHSVGLMWWMLAR |
|  |  | FTPGTFTNQIQAAFR |
|  |  | DPEEIEKEEQAAAEK |
|  |  | AIVAIENPADVSVISSR |
|  |  | EHPWEVMPDLYFYR |
|  |  | FTPGTFTNQIQAAFREPR |
|  |  | AIVAIENPADVSVISSRNTGQR |
|  |  | FLAAGTHLGGTNLDFQMEQYIYK |
|  |  | FLAAGTHLGGTNLDFQMEQYIYKR |
|  |  | ADHQPLTEASYVNLPTIALCNTDSPLR |
|  |  |  |
| 34 | polypyrimidine tract-binding protein 1 isoform | LHGKPIR |
|  |  | VTNLLMLK |
|  |  | HQNVQLPR |
|  |  | DYGNSPLHR |
|  |  | VLFSSNGGVVK |
|  |  | GQPIYIQFSNHK |
|  |  | LSLDGQNIYNACCTLR |
|  |  | VTPQSLFILFGVYGDVQR |
|  |  | KLPIDVTEGEVISLGLPFGK |
|  |  | NNQFQALLQYADPVSAQHAK |
|  |  | IAIPGLAGAGNSVLLVSNLNPER |
|  |  | ENALVQMADGNQAQLAMSHLNGHK |
|  |  | NFQNIFPPSATLHLSNIPPSVSEEDLK |
|  |  |  |
| 35 | heat shock protein 105 kDa | MFEELGQR |
|  |  | AFNDPFIQK |
|  |  | FVVQNVSAQK |
|  |  | LKETAENSLK |
|  |  | LVEHFCAEFK |
|  |  | QDLPSLDEKPR |
|  |  | VLGTAFDPFLGGK |
|  |  | NAVEEYVYEFR |
|  |  | DISTTLNADEAVAR |
|  |  | AGGIETIANEFSDR |
|  |  | VEDVSAVEIVGGATR |
|  |  | GCALQCAILSPAFK |
|  |  | EKENLSYDLVPLK |
|  |  | SQFEELCAELLQK |
|  |  | FICEQDHQNFLR |
|  |  | SVLDAAQIVGLNCLR |
|  |  | NQQITHANNTVSNFK |
|  |  | GCALQCAILSPAFKVR |
|  |  | LMNDMTAVALNYGIYK |
|  |  | VNTHGIFTISTASMVEK |
|  |  | NQQITHANNTVSNFKR |
|  |  | KPVTDCVISVPSFFTDAER |
|  |  | RGPFELEAFYSDPQGVPYPEAK |
|  |  |  |
