# Supplementary material for: Differential Proteomic Analysis of Human Erythroblasts Undergoing Apoptosis Induced by Epo-Withdrawal
Source: PLoS One. 2012 Jun 18;7(6):e38356. doi: 10.1371/journal.pone.0038356 (PMC3377639; doi:10.1371/journal.pone.0038356)
Supplement: Table S4 — lists all peptides identified by mass spectrometry from each individual spot detailed in Table 4 . (DOCX) [file pone.0038356.s007.docx]

| **Supporting information Table S4. All peptides detected.** | | |
| --- | --- | --- |
| **Spot No.** | **Identified Proteins** | **Peptides detected** |
| 23 | flavin reductase | LQAVTDDHIR |
|  |  | NDLSPTTVMSEGAR |
|  |  | TVAGQDAVIVLLGTR |
|  |  | VVACTSAFLLWDPTK |
|  |  | VVACTSAFLLWDPTKVPPR |
|  |  | LPSEGPRPAHVVVGDVLQAADVDK |
|  |  |  |
| 24 | ubiquitin-conju-gating enzyme E2 | VVLQELR |
|  |  | VVLQELRR |
|  |  | WQNSYSIK |
|  |  | YPEAPPFVR |
|  |  | PGEVQASYLK |
|  |  | SQSKLSDEGR |
|  |  | WTGMIIGPPR |
|  |  | LMMSKENMK |
|  |  | LLEELEEGQK |
|  |  | IYSLKIECGPK |
|  |  | INMNGVNSSNGVVDPR |
|  |  | AISVLAKWQNSYSIK |
|  |  | GVGDGTVSWGLEDDEDMTLTR |
|  |  |  |
| 24 | flavin reductase | LQAVTDDHIR |
|  |  | NDLSPTTVMSEGAR |
|  |  | TVAGQDAVIVLLGTR |
|  |  | VVACTSAFLLWDPTK |
|  |  | LPSEGPRPAHVVVGDVLQAADVDK |
|  |  |  |
| 25 | serine/threonine-protein phos-phatase PP1-alpha catalytic subunit | LNLDSIIGR |
|  |  | HDLDLICR |
|  |  | LLEVQGSRPGK |
|  |  | IYGFYDECK |
|  |  | YPENFFLLR |
|  |  | NVQLTENEIR |
|  |  | GVSFTFGAEVVAK |
|  |  | IYGFYDECKR |
|  |  | IKYPENFFLLR |
|  |  | QSLETICLLLAYK |
|  |  | FLHKHDLDLICR |
|  |  | AHQVVEDGYEFFAK |
|  |  | ICGDIHGQYYDLLR |
|  |  | AHQVVEDGYEFFAKR |
|  |  | YGQFSGLNPGGRPITPPR |
|  |  | EIFLSQPILLELEAPLK |
|  |  | TFTDCFNCLPIAAIVDEK |
|  |  | IFCCHGGLSPDLQSMEQIR |
|  |  | LFEYGGFPPESNYLFLGDYVDR |
|  |  |  |
| 26 | enoyl-CoA hydratase, mitochondrial | HWDHLTQVK |
|  |  | LFYSTFATDDR |
|  |  | SLAMEMVLTGDR |
|  |  | LFYSTFATDDRK |
|  |  | NNTVGLIQLNRPK |
|  |  | ESVNAAFEMTLTEGSK |
|  |  | EMQNLSFQDCYSSK |
|  |  | AQFAQPEILIGTIPGAGGTQR |
|  |  |  |
| 27 | cytochrome c oxidase subunit 4 | ESFAEMNR |
|  |  | VNPIQGLASK |
|  |  | DHPLPEVAHVK |
|  |  | ASWSSLSMDEK |
|  |  | FKESFAEMNR |
|  |  | RDHPLPEVAHVK |
|  |  | SEDFSLPAYMDR |
|  |  | HYVYGPLPQSFDK |
|  |  | SEDFSLPAYMDRR |
|  |  | ASWSSLSMDEKVELYR |
|  |  | HYVYGPLPQSFDKEWVAK |
|  |  |  |
| 28 | haloacid dehalogenase-like hydrolase domain containing 3 | LLTWDVK |
|  |  | IFQEALR |
|  |  | LAVISNFDR |
|  |  | HPLGEAYATK |
|  |  | LAVISNFDRR |
|  |  | LRHPLGEAYATK |
|  |  | AHGLEVEPSALEQGFR |
|  |  | AQSHSFPNYGLSHGLTSR |
|  |  | AVGMHSFLVVGPQALDPVVR |
|  |  | DFSHPCTWQVLDGAEDTLR |
|  |  | LAHMEPVVAAHVGDNYLCDYQGPR |
|  |  |  |
| 29 | ubiquitin-conju-gating enzyme E2 | MAGLPRR |
|  |  | ICLDILK |
|  |  | WSPALQIR |
|  |  | IYHPNVDK |
|  |  | AEPDESNAR |
|  |  | LLAEPVPGIK |
|  |  | TNEAQAIETAR |
|  |  | DKWSPALQIR |
|  |  | IYHPNVDKLGR |
|  |  | TNEAQAIETARAWTR |
|  |  | LELFLPEEYPMAAPK |
|  |  | LLAEPVPGIKAEPDESNAR |
|  |  | YFHVVIAGPQDSPFEGGTFK |
