# Supplementary material for: Differential Proteomic Analysis of Human Erythroblasts Undergoing Apoptosis Induced by Epo-Withdrawal
Source: PLoS One. 2012 Jun 18;7(6):e38356. doi: 10.1371/journal.pone.0038356 (PMC3377639; doi:10.1371/journal.pone.0038356)
Supplement: Table S5 — lists all peptides identified by mass spectrometry from each individual spot detailed in Table 5 . (DOCX) [file pone.0038356.s008.docx]

| **Supporting information Table S5 All peptides detected** | | |
| --- | --- | --- |
| **Spot No.** | **Identified proteins** | **Peptides detected** |
| 36 | clathrin light chain A | LCDFNPK |
|  |  | LEALDANSR |
|  |  | LQSEPESIR |
|  |  | ELEEWYAR |
|  |  | WREEQMER |
|  |  | LQSEPESIRK |
|  |  | AIKELEEWYAR |
|  |  | AAEEAFVNDIDESSPGTEWER |
|  |  | ANNRAAEEAFVNDIDESSPGTEWER |
|  |  |  |
| 37 | dynactin subunit 2 | GLDFSDR |
|  |  | YADLPGIAR |
|  |  | LLLQLEATK |
|  |  | LTELETAVR |
|  |  | RLLLQLEATK |
|  |  | RLTELETAVR |
|  |  | VHQLYETIQR |
|  |  | ADPKYADLPGIAR |
|  |  | LTPVLLAKQLAALK |
|  |  | WSPIASTLPELVQR |
|  |  | VSALDLAVLDQVEAR |
|  |  | DNTTLLTQVQTTMR |
|  |  | LLHEVQELTTEVEK |
|  |  | ENLATVEGNFASIDER |
|  |  | LLGPDAAINLTDPDGALAK |
|  |  | TGYESGEYEMLGEGLGVK |
|  |  | LLGPDAAINLTDPDGALAKR |
|  |  | TTGTPPDSSLVTYELHSRPEQDK |
|  |  | TTGTPPDSSLVTYELHSRPEQDKFSQAAK |
|  |  |  |
| 38 | heat shock 70 kDa protein 4 | FQESEERPK |
|  |  | CTPACISFGPK |
|  |  | AFSDPFVEAEK |
|  |  | QDLPALEEKPR |
|  |  | VLATAFDTTLGGR |
|  |  | FLEMCNDLLAR |
|  |  | NAVEEYVYEMR |
|  |  | EDIYAVEIVGGATR |
|  |  | AGGIETIANEYSDR |
|  |  | ELSTTLNADEAVTR |
|  |  | GCALQCAILSPAFK |
|  |  | FVSEDDRNSFTLK |
|  |  | LKVLATAFDTTLGGR |
|  |  | NFTTEQVTAMLLSK |
|  |  | KEDIYAVEIVGGATR |
|  |  | SVMDATQIAGLNCLR |
|  |  | EFSITDVVPYPISLR |
|  |  | LMNETTAVALAYGIYK |
|  |  | SNLAYDIVQLPTGLTGIK |
|  |  | NKEDQYDHLDAADMTK |
|  |  | VREFSITDVVPYPISLR |
|  |  | FDEVLVNHFCEEFGKK |
|  |  | VNVHGIFSVSSASLVEVHK |
|  |  | LEDTENWLYEDGEDQPK |
|  |  | TSTVDLPIENQLLWQIDR |
|  |  | KPVVDCVVSVPCFYTDAER |
|  |  | VRVNVHGIFSVSSASLVEVHK |
|  |  | NVVFVDMGHSAYQVSVCAFNR |
|  |  | VTYMEEERNFTTEQVTAMLLSK |
|  |  |  |
| 39 | polypyrimidine tract-binding protein 1 | LHGKPIR |
|  |  | VTNLLMLK |
|  |  | HQNVQLPR |
|  |  | DYGNSPLHR |
|  |  | VLFSSNGGVVK |
|  |  | DGIVPDIAVGTKR |
|  |  | GQPIYIQFSNHK |
|  |  | LSLDGQNIYNACCTLR |
|  |  | LPIDVTEGEVISLGLPFGK |
|  |  | VTPQSLFILFGVYGDVQR |
|  |  | KLPIDVTEGEVISLGLPFGK |
|  |  | EGQEDQGLTKDYGNSPLHR |
|  |  | NNQFQALLQYADPVSAQHAK |
|  |  | IAIPGLAGAGNSVLLVSNLNPER |
|  |  | IIVENLFYPVTLDVLHQIFSK |
|  |  | ENALVQMADGNQAQLAMSHLNGHK |
|  |  | NFQNIFPPSATLHLSNIPPSVSEEDLK |
|  |  | NQAFIEMNTEEAANTMVNYYTSVTPVLR |
|  |  | RGSDELFSTCVTNGPFIMSSNSASAANGNDSK |
|  |  | MALIQMGSVEEAVQALIDLHNHDLGENHHLR |
|  |  | AQAALQAVNSVQSGNLALAASAAAVDAGMAMAGQSPVLR |
|  |  |  |
| 40 | eukaryotic translation initiation factor 4H | VDIAEGRK |
|  |  | AYSSFGGGR |
|  |  | FRDGPPLR |
|  |  | SLRVDIAEGR |
|  |  | EALTYDGALLGDR |
|  |  | DDFLGGRGGSRPGDR |
|  |  | GFCYVEFDEVDSLK |
|  |  | GSNMDFREPTEEER |
|  |  | DDFNSGFRDDFLGGR |
|  |  | TVATPLNQVANPNSAIFGGARPR |
|  |  |  |
| 41 | heterogeneous nuclear ribonu-cleoprotein | GGDLMAYDR |
|  |  | VVLIGGKPDR |
|  |  | GSDFDCELR |
|  |  | NTDEMVELR |
|  |  | GGDLMAYDRR |
|  |  | NLPLPPPPPPR |
|  |  | IDEPLEGSEDR |
|  |  | IILDLISESPIK |
|  |  | LLIHQSLAGGIIGVK |
|  |  | LFQECCPHSTDR |
|  |  | RPAEDMEEEQAFK |
|  |  | ILSISADIETIGEILK |
|  |  | RPAEDMEEEQAFKR |
|  |  | TDYNASVSVPDSSGPER |
|  |  | ILSISADIETIGEILKK |
|  |  | GSYGDLGGPIITTQVTIPK |
|  |  | IITITGTQDQIQNAQYLLQNSVK |
|  |  |  |
| 42 | heterogeneous nuclear ribonu-cleoproteins C1/C2 | AAVAGEDGR |
|  |  | MYSYPAR |
|  |  | VPPPPPIAR |
|  |  | VFIGNLNTLVVK |
|  |  | GFAFVQYVNER |
|  |  | SDVEAIFSKYGK |
|  |  | QKVDSLLENLEK |
|  |  | VFIGNLNTLVVKK |
|  |  | DYYDRMYSYPAR |
|  |  | MIAGQVLDINLAAEPK |
|  |  | SAAEMYGSSFDLDYDFQR |
|  |  | RSAAEMYGSSFDLDYDFQR |
|  |  | SAAEMYGSSFDLDYDFQRDYYDR |
|  |  |  |
| 43 | HSP90 co-chaperone p23 | SILCCLR |
|  |  | GESGQSWPR |
|  |  | SILCCLRK |
|  |  | KGESGQSWPR |
|  |  | TDRSILCCLR |
|  |  | LTFSCLGGSDNFK |
|  |  | LNWLSVDFNNWK |
|  |  | SKLTFSCLGGSDNFK |
|  |  | HLNEIDLFHCIDPNDSK |
|  |  | DYVFIEFCVEDSKDVNVNFEK |
|  |  |  |
| 44 | ATP-dependent RNA helicase DDX1 | LNLSQVR |
|  |  | QNLERFK |
|  |  | FGFGFGGTGK |
|  |  | VPVDEFDGK |
|  |  | ILKGEYAVR |
|  |  | ELLIIGGVAAR |
|  |  | QNYVHRIGR |
|  |  | EWHGCRATK |
|  |  | FLICTDVAAR |
|  |  | MDQAIIFCR |
|  |  | VWYHVCSSR |
|  |  | LRELLIIGGVAAR |
|  |  | DLGLAFEIPPHMK |
|  |  | GHQFSCVCLHGDR |
|  |  | FNFGEEEFKFPPK |
|  |  | MHNQIPQVTSDGKR |
|  |  | GSAFAIGSDGLCCQSR |
|  |  | GHVDILAPTVQELAALEK |
|  |  | DQLSVLENGVDIVVGTPGR |
|  |  | GIDIHGVPYVINVTLPDEK |
|  |  | EAQTSFLHLGYLPNQLFR |
|  |  | ALIVEPSRELAEQTLNNIK |
|  |  | FLVLDEADGLLSQGYSDFINR |
|  |  |  |
| 45 | heterogeneous nuclear ribonu-cleoproteins C1/C2 | MYSYPAR |
|  |  | VPPPPPIAR |
|  |  | KSDVEAIFSK |
|  |  | VFIGNLNTLVVK |
|  |  | GFAFVQYVNER |
|  |  | SDVEAIFSKYGK |
|  |  | QKVDSLLENLEK |
|  |  | VFIGNLNTLVVKK |
|  |  | GDDQLELIKDDEK |
|  |  | MIAGQVLDINLAAEPK |
|  |  | SAAEMYGSSFDLDYDFQR |
|  |  | RSAAEMYGSSFDLDYDFQR |
|  |  |  |
| 46 | lamin-A/C | LAVYIDR |
|  |  | NIYSEELR |
|  |  | LADALQELR |
|  |  | EGDLIAAQAR |
|  |  | SLETENAGLR |
|  |  | AAYEAELGDAR |
|  |  | LVEIDNGKQR |
|  |  | KQLQDEMLR |
|  |  | TLEGELHDLR |
|  |  | LRDLEDSLAR |
|  |  | QNGDDPLLTYR |
|  |  | AAYEAELGDARK |
|  |  | SSFSQHARTSGR |
|  |  | LALDMEIHAYR |
|  |  | SLETENAGLRLR |
|  |  | GQVAKLEAALGEAK |
|  |  | LRITESEEVVSR |
|  |  | LALDMEIHAYRK |
|  |  | VAVEEVDEEGKFVR |
|  |  | TALINSTGEEVAMRK |
|  |  | LQEKEDLQELNDR |
|  |  | TLEGELHDLRGQVAK |
|  |  | IRIDSLSAQLSQLQK |
|  |  | NSNLVGAAHEELQQSR |
|  |  | QNGDDPLLTYRFPPK |
|  |  |  |
| 48 | heterogeneous nuclear ribonu-cleoprotein K | DLAGSIIGK |
|  |  | GGDLMAYDR |
|  |  | VVLIGGKPDR |
|  |  | GSDFDCELR |
|  |  | NTDEMVELR |
|  |  | GGDLMAYDRR |
|  |  | NLPLPPPPPPR |
|  |  | IDEPLEGSEDR |
|  |  | IILDLISESPIK |
|  |  | LLIHQSLAGGIIGVK |
|  |  | LFQECCPHSTDR |
|  |  | RPAEDMEEEQAFK |
|  |  | ILSISADIETIGEILK |
|  |  | RPAEDMEEEQAFKR |
|  |  | TDYNASVSVPDSSGPER |
|  |  | ILSISADIETIGEILKK |
|  |  | GSYGDLGGPIITTQVTIPK |
|  |  | IITITGTQDQIQNAQYLLQNSVK |
|  |  |  |
| 49 | alanyl-tRNA synthetase | FIDFFK |
|  |  | FIDFFKR |
|  |  | AVFDETYPDPVR |
|  |  | AEEIANEMIEAAK |
|  |  | DIINEEEVQFLK |
|  |  | VGAEDADGIDMAYR |
|  |  | GLVVDMDGFEEER |
|  |  | VGDQVWLFIDEPR |
|  |  | GGYVLHIGTIYGDLK |
|  |  | GLVVDMDGFEEERK |
|  |  | ITCLCQVPQNAANR |
|  |  | TITVALADGGRPDNTGR |
|  |  | NSSHAGAFVIVTEEAIAK |
|  |  | MALELLTQEFGIPIER |
|  |  | AVYTQDCPLAAAKAIQGLR |
|  |  | GAGGEDLIMLDIYAIEELR |
|  |  | RPIMSNHTATHILNFALR |
|  |  | NVGCLQEALQLATSFAQLR |
|  |  | MSNYDTDLFVPYFEAIQK |
|  |  | RRPIMSNHTATHILNFALR |
|  |  | YNYHLDSSGSYVFENTVATVMALR |
|  |  |  |
| 50 | Heterogeneous nuclear ribonu-cleoprotein K | GGDLMAYDR |
|  |  | VVLIGGKPDR |
|  |  | GSDFDCELR |
|  |  | NTDEMVELR |
|  |  | GGDLMAYDRR |
|  |  | NLPLPPPPPPR |
|  |  | IDEPLEGSEDR |
|  |  | IILDLISESPIK |
|  |  | LLIHQSLAGGIIGVK |
|  |  | LFQECCPHSTDR |
|  |  | RPAEDMEEEQAFK |
|  |  | RPAEDMEEEQAFKR |
|  |  | TDYNASVSVPDSSGPER |
|  |  | ILSISADIETIGEILKK |
|  |  | GSYGDLGGPIITTQVTIPK |
|  |  | IITITGTQDQIQNAQYLLQNSVK |
|  |  |  |
| 51 | acylamino-acid-releasing enzyme | VVFDSAQR |
|  |  | LGIRFCTNR |
|  |  | TVHTEWTQR |
|  |  | VTSVVVDVVPR |
|  |  | MGFAVLLVNYR |
|  |  | SALYYVDLIGGK |
|  |  | TPLLLMLGQEDR |
|  |  | CELLSDDSLAVSSPR |
|  |  | QVLLSEPEEAAALYR |
|  |  | GSTGFGQDSILSLPGNVGHQDVK |
|  |  | QPALSAACLGPEVTTQYGGQYR |
|  |  | QYLVFHDGDSVVFAGPAGNSVETR |
|  |  |  |
| 52 | serine/threonine-protein kinase PAK 2 | YLSFTPPEK |
|  |  | ELLQHPFLK |
|  |  | FYDSNTVKQK |
|  |  | KELIINEILVMK |
|  |  | LAKPLSSLTPLIMAAK |
|  |  | LTDFGFCAQITPEQSK |
|  |  | SVIDPVPAPVGDSHVDGAAK |
|  |  | ALYLIATNGTPELQNPEK |
|  |  | LTDFGFCAQITPEQSKR |
|  |  | STMVGTPYWMAPEVVTRK |
|  |  | ECLQALEFLHANQVIHR |
|  |  | IGQGASGTVFTATDVALGQEVAIK |
|  |  | YLSFTPPEKDGFPSGTPALNAK |
|  |  | GTEAPAVVTEEEDDDEETAPPVIAPRPDHTK |
|  |  |  |
| 53 | elongation factor  1-beta | LEECVR |
|  |  | WYNHIK |
|  |  | LVPVGYGIK |
|  |  | SIQADGLVWGSSK |
|  |  | SPAGLQVLNDYLADK |
|  |  | YGPADVEDTTGSGATDSK |
|  |  | SSILLDVKPWDDETDMAK |
|  |  | SYIEGYVPSQADVAVFEAVSSPPPADLCHALR |
