# Supplementary material for: Differential Proteomic Analysis of Human Erythroblasts Undergoing Apoptosis Induced by Epo-Withdrawal
Source: PLoS One. 2012 Jun 18;7(6):e38356. doi: 10.1371/journal.pone.0038356 (PMC3377639; doi:10.1371/journal.pone.0038356)
Supplement: Table S6 — lists all peptides identified by mass spectrometry from each individual spot detailed in Table 6 . (DOCX) [file pone.0038356.s009.docx]

| **Supporting Information Table S6. All peptides** | | |
| --- | --- | --- |
| **Spot No.** | **Identified proteins** | **Peptides detected** |
| 1 | Nascent poly-peptide-associated complex alpha subunit isoform b | DIELVMSQANVSR |
|  |  | SPASDTYIVFGEAK |
|  |  | NILFVITKPDVYK |
|  |  | IEDLSQQAQLAAAEK |
|  |  | SKNILFVITKPDVYK |
|  |  |  |
| 2 | NDUFS3 NADH dehydrogenase (ubi-quinone) Fe-S protein | AANWYER |
|  |  | DHTNAQFK |
|  |  | DFPLSGYVELR |
|  |  | FEIVYNLLSLR |
|  |  | SLVDLTAVDVPTR |
|  |  | KDFPLSGYVELR |
|  |  | VVAEPVELAQEFR |
|  |  | ESAGADTRPTVRPR |
|  |  | ILTDYGFEGHPFR |
|  |  | VVAEPVELAQEFRK |
|  |  | QLSAFGEYVAEILPK |
|  |  | ILTDYGFEGHPFRK |
|  |  | FDLNSPWEAFPVYR |
|  |  | QNRFEIVYNLLSLR |
|  |  | SLVDLTAVDVPTRQNR |
|  |  | KFDLNSPWEAFPVYR |
|  |  | TYTDELTPIESAVSVFK |
|  |  | EIWDMFGVFFANHPDLR |
|  |  |  |
| 2 | Heat shock 27kDa protein | VPFSLLR |
|  |  | HTADRWR |
|  |  | DWYPHSR |
|  |  | GPSWDPFR |
|  |  | QDEHGYISR |
|  |  | LFDQAFGLPR |
|  |  | AQLGGPEAAKSDETAAK |
|  |  | VSLDVNHFAPDELTVK |
|  |  | LATQSNEITIPVTFESR |
|  |  |  |
| 2 | Lamin A/C | VREEFK |
|  |  | LAVYIDR |
|  |  | NIYSEELR |
|  |  | EGDLIAAQAR |
|  |  | QLQDEMLR |
|  |  | SLETENAGLR |
|  |  | LAVYIDRVR |
|  |  | EDLQELNDR |
|  |  | ITESEEVVSR |
|  |  | AAYEAELGDAR |
|  |  | KQLQDEMLR |
|  |  | TLEGELHDLR |
|  |  | AAYEAELGDARK |
|  |  | LQTMKEELDFQK |
|  |  | LQEKEDLQELNDR |
|  |  | TLEGELHDLRGQVAK |
|  |  |  |
| 3 | purine nucleoside phosphorylase | LVFGFLNGR |
|  |  | VFGFSLITNK |
|  |  | FPAMSDAYDR |
|  |  | FEVGDIMLIR |
|  |  | FHMYEGYPLWK |
|  |  | FGDRFPAMSDAYDR |
|  |  | DHINLPGFSGQNPLR |
|  |  | LGADAVGMSTVPEVIVAR |
|  |  | LEQFVSILMASIPLPDK |
|  |  | HRPQVAIICGSGLGGLTDK |
|  |  | LTQAQIFDYGEIPNFPR |
|  |  | LEQFVSILMASIPLPDKAS |
|  |  | VFHLLGVDTLVVTNAAGGLNPK |
|  |  | DHINLPGFSGQNPLRGPNDER |
|  |  | QAAQKLEQFVSILMASIPLPDK |
|  |  | ELQEGTYVMVAGPSFETVAECR |
|  |  |  |
| 4 | heat shock protein  HSP 90-alpha | ELHINLIPNK |
|  |  | ADLINNLGTIAK |
|  |  | DCPAQSAEYPR |
|  |  | EDQTEYLEER |
|  |  | EFEGKTLVSVTK |
|  |  | TLTIVDTGIGMTK |
|  |  | ELISNSSDALDKIR |
|  |  | ELHINLIPNKQDR |
|  |  | QDRTLTIVDTGIGMTK |
|  |  | HSQFIGYPITLFVEK |
|  |  | KHSQFIGYPITLFVEK |
|  |  | VILHLKEDQTEYLEER |
|  |  | MPPCSGGDGSTPPGPSLRDR |
|  |  | HNDDEQYAWESSAGGSFTVR |
|  |  | QSQGAGQHLYKDLQPFILLR |
|  |  |  |
| 5 | heat shock protein  HSP 90-alpha | ELHINLIPNK |
|  |  | ADLINNLGTIAK |
|  |  | DCPAQSAEYPR |
|  |  | EDQTEYLEER |
|  |  | TLTIVDTGIGMTK |
|  |  | ELISNSSDALDKIR |
|  |  | ELHINLIPNKQDR |
|  |  | HSQFIGYPITLFVEK |
|  |  | KHSQFIGYPITLFVEK |
|  |  | VILHLKEDQTEYLEER |
|  |  | HSQFIGYPITLFVEKER |
|  |  | HNDDEQYAWESSAGGSFTVR |
|  |  |  |
| 6 | heat shock protein  HSP 90-beta | ADHGEPIGR |
|  |  | IDIIPNPQER |
|  |  | APFDLFENKK |
|  |  | ADLINNLGTIAK |
|  |  | EISDDEAEEEK |
|  |  | IRYESLTDPSK |
|  |  | EDQTEYLEER |
|  |  | TLTLVDTGIGMTK |
|  |  | ELISNASDALDKIR |
|  |  | ELKIDIIPNPQER |
|  |  | HSQFIGYPITLYLEK |
|  |  | KHLEINPDHPIVETLR |
|  |  | KHSQFIGYPITLYLEK |
|  |  | VILHLKEDQTEYLEER |
|  |  | HSQFIGYPITLYLEKER |
|  |  | HNDDEQYAWESSAGGSFTVR |
|  |  |  |
| 7 | heat shock protein  HSP 90-beta | ADHGEPIGR |
|  |  | IDIIPNPQER |
|  |  | APFDLFENKK |
|  |  | ADLINNLGTIAK |
|  |  | EISDDEAEEEK |
|  |  | EDQTEYLEER |
|  |  | TLTLVDTGIGMTK |
|  |  | ELISNASDALDKIR |
|  |  | ELKIDIIPNPQER |
|  |  | HSQFIGYPITLYLEK |
|  |  | KHSQFIGYPITLYLEK |
|  |  | VILHLKEDQTEYLEER |
|  |  | HSQFIGYPITLYLEKER |
|  |  | HNDDEQYAWESSAGGSFTVR |
|  |  |  |
| 8 | acidic leucine-rich nuclear phosphoprotein 32 family member | NRTPAAVR |
|  |  | RIHLELR |
|  |  | KLELSENR |
|  |  | ELVLDNCK |
|  |  | DISTLEPLK |
|  |  | DISTLEPLKK |
|  |  | IFGGLDMLAEK |
|  |  | LPNLTHLNLSGNK |
|  |  | LLPQLTYLDGYDR |
|  |  | SLDLFNCEVTNLNDYR |
|  |  |  |
| 9 | 60S acidic ribosomal protein P0 | GNVGFVFTK |
|  |  | IIQLLDDYPK |
|  |  | GHLENNPALEK |
|  |  | CFIVGADNVGSK |
|  |  | TSFFQALGITTK |
|  |  | AVVLMGKNTMMR |
|  |  | GTIEILSDVQLIK |
|  |  | EDLTEIRDMLLANK |
|  |  | GNVGFVFTKEDLTEIR |
|  |  | VLALSVETDYTFPLAEK |
|  |  | AGAIAPCEVTVPAQNTGLGPEK |
|  |  | AFLADPSAFVAAAPVAAATTAAPAAAAAPAK |
