# Supplementary material for: Differential Proteomic Analysis of Human Erythroblasts Undergoing Apoptosis Induced by Epo-Withdrawal
Source: PLoS One. 2012 Jun 18;7(6):e38356. doi: 10.1371/journal.pone.0038356 (PMC3377639; doi:10.1371/journal.pone.0038356)
Supplement: Table S7 — lists all peptides identified by mass spectrometry from each individual spot detailed in Table 7 . (DOCX) [file pone.0038356.s010.docx]

| **Supporting information Table S7. All peptides detected.** | | |
| --- | --- | --- |
| **Spot No.** | **Identified protein** | **Peptides detected** |
| 1 | Matrin-3 isoform a | GPGPLQER |
|  |  | ALWFQGR |
|  |  | DGSASAAAKK |
|  |  | EPPYRVPR |
|  |  | SFQQSSLSR |
|  |  | IKNYILMR |
|  |  | GPLPLSSQHR |
|  |  | VVHIMDFQR |
|  |  | TEEGPTLSYGR |
|  |  | FDSEYERMGR |
|  |  | GNLGAGNGNLQGPR |
|  |  | RTEEGPTLSYGR |
|  |  | DLDELSRYPEDK |
|  |  | EWSQHINGASHSR |
|  |  | ITPENLPQILLQLK |
|  |  | GPSLNPVLDYDHGSR |
|  |  | ITPENLPQILLQLKR |
|  |  | GAPPSSNIEDFHGLLPK |
|  |  | GDADQASNILASFGLSAR |
|  |  | RGAPPSSNIEDFHGLLPK |
|  |  | IGPYQPNVPVGIDYVIPK |
|  |  | CRDDSFFGETSHNYHK |
|  |  | VIHLSNLPHSGYSDSAVLK |
|  |  | DSFDDRGPSLNPVLDYDHGSR |
|  |  | DLSAAGIGLLAAATQSLSMPASLGR |
|  |  |  |
| 2 | Nucleolin | VFGNEIK |
|  |  | LELQGPR |
|  |  | ESFDGSVR |
|  |  | TGISDVFAK |
|  |  | GIAYIEFK |
|  |  | NDLAVVDVR |
|  |  | EALNSCNKR |
|  |  | SISLYYTGEK |
|  |  | EVFEDAAEIR |
|  |  | IVTDRETGSSK |
|  |  | ATFIKVPQNQNGK |
|  |  | GFGFVDFNSEEDAK |
|  |  | GYAFIEFASFEDAK |
|  |  | ALELTGLKVFGNEIK |
|  |  | FGYVDFESAEDLEK |
|  |  | VTLDWAKPKGEGGFGGR |
|  |  | KFGYVDFESAEDLEK |
|  |  | SKGYAFIEFASFEDAK |
|  |  | GLSEDTTEETLKESFDGSVR |
|  |  | VEGTEPTTAFNLFVGNLNFNK |
|  |  | TLVLSNLSYSATEETLQEVFEK |
|  |  |  |
| 3 | Splicing Factor 1 | ILRPWQSSETR |
|  |  | ATGANATPLDFPSK |
|  |  | SPSPEPIYNSEGK |
|  |  | MATGANATPLDFPSK |
|  |  | AYIVQLQIEDLTR |
|  |  | QGIETPEDQNDLRK |
|  |  | TVIPGMPTVIPPGLTR |
|  |  | TVIPGMPTVIPPGLTREQER |
|  |  | HNLITEMVALNPDFKPPADYKPPATR |
|  |  |  |
| 4 | Eukaryotic translation initiation factor 4E type 2 | WIIRLR |
|  |  | MPGRLGPQR |
|  |  | FQEDIISIWNK |
|  |  | VLNLPPNTIMEYK |
|  |  | QIGTFASVEQFWR |
|  |  | TPGRPTSSQSYEQNIK |
|  |  | RTPGRPTSSQSYEQNIK |
|  |  | AVVPGPAEHPLQYNYTFWYSR |
|  |  | FYSHMVRPGDLTGHSDF HLFK |
|  |  |  |
| 4 | Lysophospholipase-like 1 | CIVSPAGR |
|  |  | IIYPTAPPR |
|  |  | ASAVYQALQK |
|  |  | MAAASGSVLQR |
|  |  | GGISNVWFDR |
|  |  | QVLNQDLTFQHIK |
|  |  | FHSFPNVYHELSK |
|  |  | NHQDVAGVFALSSFLNK |
|  |  | HSASLIFLHGSGDSGQGLR |
